# Synthesis, Kinetic and Conformational Studies of 2-Substituted-5-(β-d-glucopyranosyl)-pyrimidin-4-ones as Potential Inhibitors of Glycogen Phosphorylase

**DOI:** 10.3390/molecules25225463

**Published:** 2020-11-22

**Authors:** Konstantinos F. Mavreas, Dionysios D. Neofytos, Evangelia D. Chrysina, Alessandro Venturini, Thanasis Gimisis

**Affiliations:** 1 Laboratory of Organic Chemistry, Department of Chemistry, National and Kapodistrian University of Athens, 15784 Athens, Greece; kmavreas@chem.uoa.gr; 2 Institute of Chemical Biology, National Hellenic Research Foundation, 11635 Athens, Greece; dneofytos@chem.uoa.gr; 3 Istituto ISOF, Consiglio Nazionale delle Ricerche, 40129 Bologna, Italy

**Keywords:** type 2 diabetes, cancer, glycogen phosphorylase, catalytic site inhibitors, 5-(β-d-glucopyranosyl)pyrimidin-4-one synthesis, DFT conformational analysis

## Abstract

Dysregulation of glycogen phosphorylase, an enzyme involved in glucose homeostasis, may lead to a number of pathological states such as type 2 diabetes and cancer, making it an important molecular target for the development of new forms of pharmaceutical intervention. Based on our previous work on the design and synthesis of 4-arylamino-1-(β-d-glucopyranosyl)pyrimidin-2-ones, which inhibit the activity of glycogen phosphorylase by binding at its catalytic site, we report herein a general synthesis of 2-substituted-5-(β-d-glucopyranosyl)pyrimidin-4-ones, a related class of metabolically stable, *C-*glucosyl-based, analogues. The synthetic development consists of a metallated heterocycle, produced from 5-bromo-2-methylthiouracil, in addition to protected d-gluconolactone, followed by organosilane reduction. The methylthio handle allowed derivatization through hydrolysis, ammonolysis and arylamine substitution, and the new compounds were found to be potent (μM) inhibitors of rabbit muscle glycogen phosphorylase. The results were interpreted with the help of density functional theory calculations and conformational analysis and were compared with previous findings.

## 1. Introduction

The glycogen phosphorylase (GP) enzyme plays a key role in glucose homeostasis in mammals through the catabolism of glycogen to readily available glucose. Dysregulation of the liver isoform of GP occurring in type 2 diabetes has long been known to significantly contribute to abnormally high blood sugar levels, making it a molecular target for new antidiabetic agents [1,2,3]. The discovery of the expression of all isoforms of GP in cancerous tissues [4,5,6,7] is illustrated well in the Human Protein Atlas (PYGM, PYGL and PYGB isoforms [8]). Apart from providing an energy source, GP expression has been reported to be associated with activation of the pentose phosphate pathway (PPP), decreased levels of reactive oxygen species (ROS) and cell proliferation [9]. These findings have led to a number of works on new possible lines of cancer treatment [10,11,12,13]. Over the past decade, there has also been renewed interest in elucidating the exact function of the brain isoform of GP expressed in astrocytes [14]. Long thought to be an evolutionary remnant, the role of brain-GP dysregulation in impaired learning and memory formation, some types of epilepsy and even Alzheimer’s disease has dispelled theories about the possible vestigiality of glycogen presence in the central nervous system [15,16]. Due to the ubiquitous presence of GP, the design and synthesis of GP activity-modulating agents is currently an exciting field in chemical research.

**Figure 1:**
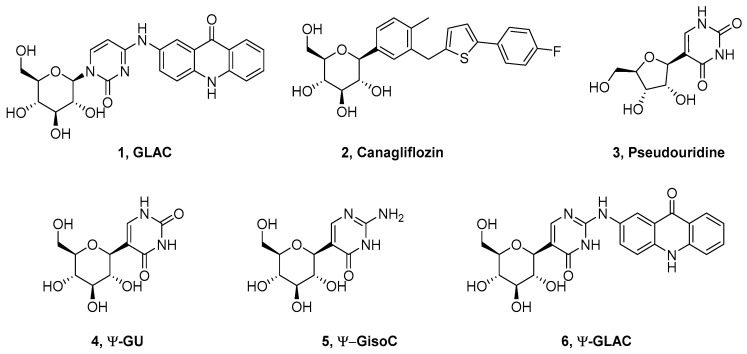
Known and target *N*- and *C*-glycosyl compounds.

The affinity of the GP catalytic site for d-glucose, which weakly inhibits glycogenolytic activity in its own right, has led to the discovery of numerous glucose-derived molecules with ever increasing inhibitory activity [17,18,19,20,21,22,23,24]. Our work on 4-arylamino-1-(β-d-glucopyranosyl)pyrimidin-2-one inhibitors, which act on the catalytic site of rabbit muscle GP (RMGP), has recently led to new molecules which exhibit RMGP inhibition in the nanomolar range, the strongest being 1-(β-d-glucopyranosyl)-4-[(acridin-9-on-2-yl)amino]pyrimidin-2-one (GLAC, **1**, Figure 1) with a Ki of 71 nM [25]. Our attention has recently shifted towards the design of metabolically stable analogues [26] of the previously synthesized inhibitors.

*C*-Glucosyl-based drugs have developed into important additions to the arsenal against type 2 diabetes, in the form of gliflozins [27]. They inhibit the renal type 2 sodium dependent glucose transporter (SGLT-2), showing strong hypoglycaemic action and offering superior half-life in comparison to classic glycosides. The most prominent example is perhaps canagliflozin (**2**, Figure 1), first approved for use in clinical practice in 2014 [28]. Indeed, *C*-glucopyranosyl derivatives have been comprehensively studied as potential GP inhibitors by Somsák and coworkers [29], leading to some of the most potent catalytic-site inhibitors known to date [26,30]. Based on our previous research [19,25,31], we decided to extend our work into the family of *C*-glucopyranosyl, nucleoside-type, molecules. Our synthetic targets included the 5-(β-d-glucopyranosyl)-derivatives of pyrimidin-2,4-one **4** (Ψ-GU) and 2-amino-pyrimidin-4-one **5** (Ψ-GisoC), glucose-analogues of the naturally occurring *C-*nucleoside pseudouridine (ΨU, **3**), as well as the *C*-glucopyranosyl analogue of GLAC (Ψ-GLAC, **6**, Figure 1). The main goal, as already mentioned, was the design of a synthetic scheme leading to more metabolically stable, strong inhibitors acting at the catalytic site of RMGP. Our synthetic plan required an activating group at the 2*-*position of the pyrimidine ring, which could be either converted to **4** and **5**, or allowed to react with arylamines towards Ψ-GLAC (**6**). Herein we describe the successful substitution of the fragile *C-N* glycosidic linkage with a more stable *C-C* bond, through the synthesis of perbenzylated and peracetylated 5-(β-d-glucopyranosyl)-2-(methylthio) pyrimidin-4-one.

## 2. Results

### 2.1. Synthesis

**Scheme 1:**
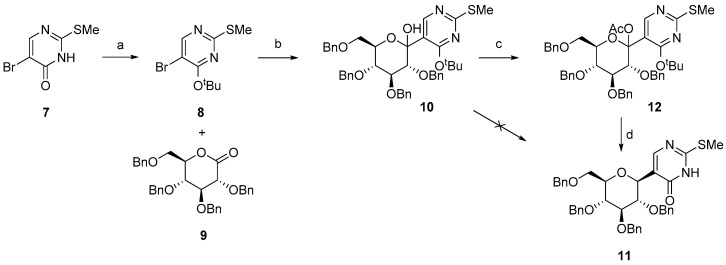
(a) (i) POCl_3_, 120 °C, 3 h, (ii) KO^t^Bu, THF, rt, 24 h, 90% over 2 steps. (b) (i) **8**, iPrMgCl·LiCl, THF, −22 °C, 4 h, (ii) **9**, THF, rt, 16 h, 74%. (c) NaH, Ac_2_O, THF, 0 °C—rt, 96 h, quantitative. (d) Et_3_SiH, TMSOTf, DCM, rt, 1 h, 78%.

Numerous methods for the synthesis of *C*-glucosyl compounds have been developed over the past decades (for a comprehensive review, see [32]). The electron-poor nature of the pyrimidine ring, as well as the desired β-(d)-configuration of the product, led to the selection of the most widely adopted method in the field of *C*-glycosylation [33,34]. It entails the coupling of 2,3,4,6-tetra-*O*-benzyl-d-glucono-1,5-lactone (**9**, Scheme 1), with a suitable metalorganic derivative of the desired pyrimidine **8**, followed by stereoselective trialkylsilane-induced reductive cleavage of the anomeric hydroxyl group in **10**. Initial attempts of utilizing silylated derivatives of d-gluconolactone, such as trimethylsilyl [35] or triethylsilyl [36], proved unsatisfactory in terms of yield and purity during coupling.

The previously unreported pyrimidine derivative **8** was synthesized from the known 5-bromo-2-(methylthio)pyrimidin-4-one (**7**) [37,38], by chlorination of position 4, with phosphorus oxychloride followed by substitution with potassium t*ert*-butoxide (Scheme 1). The methylthio group in **8** plays the role of a useful synthetic handle for further derivatization after coupling and reduction. We started our coupling attempts by lithiation of pyrimidine **8**, utilizing *n*-BuLi in a bromide-lithium exchange reaction, the same protocol that was successfully applied in the synthesis of Ψ-U(**3**) by Hanessian and Machaalani [39]. The ensuing coupling attempts with lactone **9** led to varying yields of product **10** (20–40%), and often difficult-to-purify reaction mixtures. These low yields most probably were caused by the reduced reactivity of lactone **9**, compared to ribonolactone derivatives, combined with the propensity of lithio-pyrimidines to dimerize under the reaction conditions [40].

Following extensive work by Knochel and coworkers on the use of pyrimidines as organometallic nucleophiles [41,42], we decided to switch to the turbo-Grignard reagent (iPrMgCl·LiCl in THF). To our delight, after only our first attempt we were greeted by vastly improved yields, and the purity profile allowed the purification of **10** by simple trituration. Yields were consistently high (>70%) and allowed the trouble-free scaling-up of the reaction to up to 20 mmol. The lower reactivity of Grignard reagents compared to lithiated derivatives also simplified the experimental procedure by allowing the use of a relatively concentrated reaction mixture and not requiring extremely low temperatures. Intermediate **10** was isolated as a single anomer, most probably with the hydroxyl-group in the axial position, as dictated by the anomeric effect.

In the next step, the reductive cleavage of the anomeric hydroxyl group of **10** was attempted. There are numerous literature examples in related systems [33,34,43,44], which utilize the combination of an organosilane (most commonly triethylsilane) and a Lewis acid (usually BF_3_·Et_2_O) to directly reduce the anomeric hydroxyl group. The above protocols have been reported to predominantly lead to β-stereoselective reduction of the anomeric position. In our hands, these conditions failed to produce the desired compound **11**. The only observed reaction was the acid-catalyzed cleavage of the 4-*O-tert*-butyl-group on the pyrimidine ring, as witnessed by mass spectrometry. Raising the temperature in the presence of BF_3_·Et_2_O or TMSOTf, or using stronger Lewis acids (TiCl_4_, SnCl_4_), yielded complex mixtures. Methylation of the hydroxyl group using methyl iodide, prior to reduction under the above conditions, was similarly unsuccessful. Derivatization into the corresponding phenylthionoformate ester was successful, but attempted radical deoxygenation under the Barton–McCombie conditions failed [45].

We theorized that the pyrimidine might be destabilizing the intermediate oxocarbenium cation by inductively withdrawing electron density from the anomeric carbon in the pyranose ring. Similar problems have been described in the literature for electron-poor *C*-glycosyl compounds [46,47]. In these cases, this issue was remedied by acetylating the anomeric hydroxyl group before attempting reduction. Acetylation of the hindered hydroxyl group of **10** proved rather challenging. We tried several methods (e.g., Ac_2_O/pyridine, Ac_2_O/Sc(OTf)_3,_ AcCl/DMAP-NaH), which led to either low yields or decomposition of the substrate. In the end, we found that the rather unusual combination of NaH/Ac_2_O led, after a 96 h reaction, to practically quantitative conversion into the acetylated derivative **12**, in excellent purity. Compound **12** was successfully reduced to the target compound **11** in the presence of triethylsilane and TMSOTf (Scheme 1). The desired β-(d)-configuration of the product was confirmed by observing a *J*_H1’,H2’_ = 9.5 Hz coupling constant, indicating a trans-diaxial orientation of the two hydrogen atoms. The considerable crowding of the ^1^H-NMR signals did not allow the application of NOE spectroscopy. It should be noted that attempted deprotection of the benzyl groups of intermediate **11** by hydrogenolysis (Pd(OH)_2_/C, in methanol or DMF), was unsuccessful, most probably due to a poisoning effect of the thioether group on the catalyst.

**Scheme 2:**
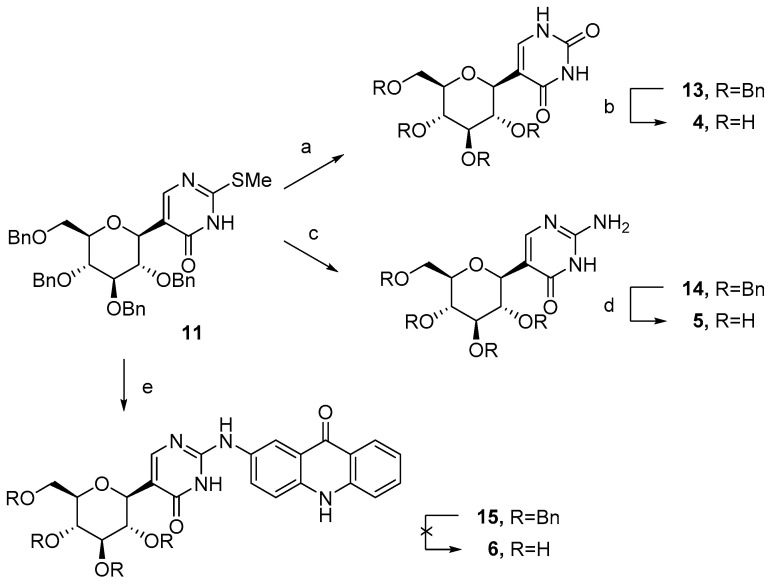
(a) H_2_O_2_, AcOH, rt-70 °C, 3 h, 87%. (b) Pd/C, H_2,_ 5% DCM in MeOH, rt, 24 h, quantitative. (c) CH_3_COONH_4_, 140 °C, 16 h, 78%. (d) Pd/C, Pd(OH)_2_/C, H_2_, 5% DCM in MeOH, rt, 96 h, 60%. (e) 2-aminoacridin-9(10*H*)-one, PivOH, 120 °C, 16 h, 61%.

We proceeded to the derivatization of intermediate **11** at position 2 of the pyrimidine ring. The thiomethyl group was successfully hydrolyzed using aqueous peracetic acid [48] to perbenzylated 5*-*(β-d-glucopyranosyl)pyrimidin-2,4-dione **13**, which was smoothly deprotected under standard hydrogenolysis conditions (Scheme 2) to yield the final product Ψ-GU (**4**) quantitatively. 

Initial attempts to directly substitute the methylthio group of **11**, by ammonia, with the aim of synthesizing the corresponding 2-aminopyrimidin-4-one (isocytosine) derivative **14**, proved to be more challenging**.** We successfully oxidized the thiomethyl group to the corresponding methylsulfone utilizing DMDO [49], but again substitution by ammonia proved unsuccessful. Ammonolysis was finally achieved by fusion of **11** with molten ammonium acetate [50], which successfully yielded the desired product **14**. This substrate proved recalcitrant to hydrogenolysis of the benzyl groups under standard conditions. Deprotection was achieved by hydrogenation, using a combination of palladium/palladium hydroxide on carbon (1/1) [51] in stoichiometric amount, which yielded the debenzylated product Ψ-GisoC (**5**) in 60% yield, after chromatographic purification. We believe that deactivation of the catalyst due to complexation of palladium with the guanidine-like moiety could explain this lack of reactivity.

In the final part of the synthesis we wanted to demonstrate that substitution of the methylthio group could also be affected by arylamines, in continuation of our previous work [19,25,31]. For this purpose, we adopted the method published by Maddess et al. [52], in which pivalic acid is used as both the reaction solvent and acid catalyst. We showcased the applicability of this method to our system, by the synthesis of the perbenzylated *C*-glucopyranosyl derivative **15**, an analogue of GLAC (**1**) [25], one of the strongest known inhibitors for the catalytic site of GP.

While substitution of the methylthio group of **11** with 2-aminoacridin-9(10*H*)-one smoothly provided intermediate **15** in good yield (Scheme 2), the intermediate **15** proved impossible to debenzylate by hydrogenolysis, even though a variety of debenzylation conditions were tested (Pd/C or Pd/C, Pd(OH)_2_/C with H_2_ under ambient, as well as increased pressures of up to 2 bar or transfer hydrogenation in DMF using Pd/C and ammonium formate). While the benzyl groups had proven to be very useful synthetically, up to this point of the synthetic scheme (due to stability, ease of handling, good solubility and facile purification by recrystallization of most intermediates), a better solution was deemed necessary. 

**Scheme 3:**
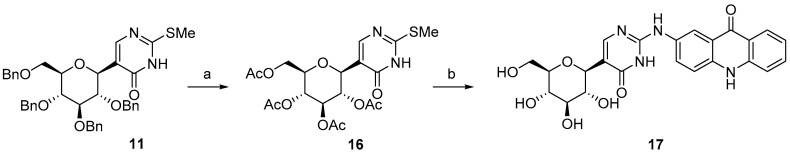
(a) Ac_2_O, TMSOTf, DCM, 0 °C–rt, 24 h, 78%. (b) (i) 2-aminoacridin-9(10*H*)-one, PivOH, 140 °C, 4 h, (ii) NH_3_, MeOH, rt, 16 h, 54%.

Ultimately, the application of a slight modification of the work by Vasella and Alzeer [53] proved to be the solution. The described method allowed for the direct conversion of the benzyl ethers of **11** to yield peracetylated compound **16** (Scheme 3). Thus, the reaction of **11** with an excess of TMSOTf and Ac_2_O led to a smooth exchange of the benzyl groups in intermediate **11** with the acetyl groups in **16**, in 78% yield. Intermediate **16** allowed for the acquisition of good quality NOESY spectra. The observed NOESY signals between *H-1’* and *H-3’* or *H-5’*, unequivocally further supported the desired β-configuration at *C-1’* of compound **16**.

The new key intermediate **16** successfully reacted with 2-aminoacridin-9(10*H*)-one, under the previously described conditions, to yield the corresponding acetylated Ψ-GLAC **17**, which exhibited a lack of solubility in a variety of solvents. For this reason, the crude product **17** was used directly in the following deprotection step in the presence of a methanolic ammonia solution. Taking advantage of a similar lack of solubility of **6**, the product was purified by repeated trituration (ethyl acetate and methanol) and centrifugation steps to yield the pure deprotected target compound **6**, with 83% yield.

### 2.2. Enzyme Kinetics

The potency of compounds Ψ-GU (**4**), Ψ-GisoC (**5**) and Ψ-GLAC (**6**) to act as inhibitors of RMGP was evaluated by kinetic studies, following previously described protocols [54]. We determined IC_50_ values of 88.5 ± 4.3, 395.7 ± 10.8 and 5.4 ± 0.5 μM for **4–6**, respectively. These values indicate that all compounds act as inhibitors of RMGP with, as expected, compound **6** exhibiting the strongest inhibition.

## 3. Discussion

**Figure 2:**
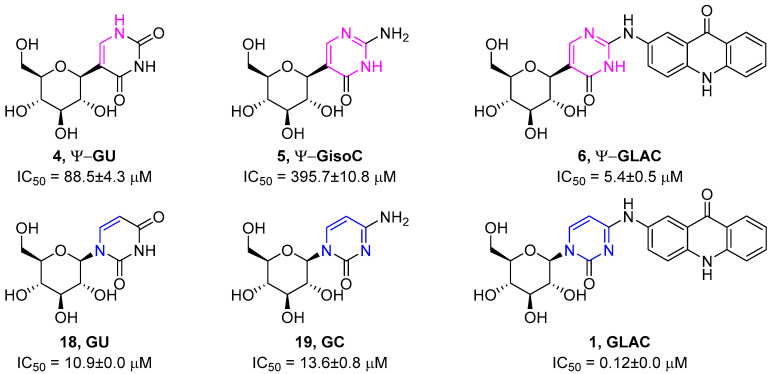
Measured values of the half maximal inhibitory concentration (IC_50_) for **4**–**6** and comparison with known derivatives. The structural changes between the new *C*- and the reported *N*-glycosyl RMGP inhibitors are indicated in magenta and blue.

We compared the kinetic results of Ψ-GU (**4**), Ψ-GisoC (**5**) and Ψ-GLAC (**6**) with the inhibitory potency of the corresponding *N-*glycosidic 1-(β-d-glucopyranosyl)pyrimidin-2-one derivatives GU (**18**), GC (**19**) and GLAC (**1**) that we have previously measured [19,25], as shown in Figure 2. From this comparison, it can be seen that Ψ-GU exhibits ~8 times weaker inhibition than GU, Ψ-GisoC is ~29 times weaker than GC (**19**), and Ψ-GLAC is ~45 times weaker than GLAC [25].

**Figure 3:**
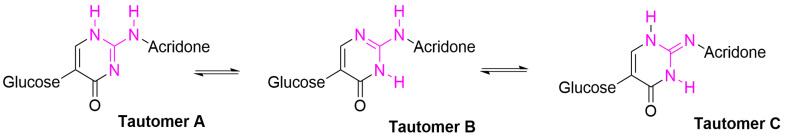
Possible tautomeric forms of Ψ-GLAC (**A**, **B** and **C**) that may affect the dihedral angle between the two chromophores.

**Figure 4:**
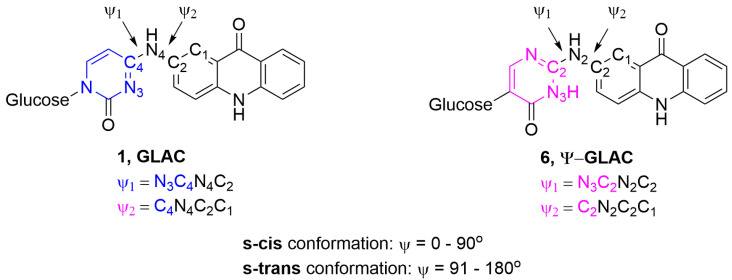
Definition of dihedral angles ψ_1_ and ψ_2_ and conformations in GLAC and Ψ-GLAC.

The profound difference in inhibitory activity between Ψ-GLAC (**6**) and GLAC (**1**) was unexpected, since docking studies with the GLIDE algorithm of the Schrödinger software package, that were performed prior to initiating the synthesis of Ψ-GLAC, had predicted an IC_50_ of ~0.2 μM (data not shown). This prompted us to undertake an alternative theoretical conformational study in an attempt to reason for the observed differences. In the case of GLAC, we have reported a detailed theoretical conformational study in the ground and excited states in order to explain GLAC’s observed photochemistry [55]. We decided to extend this study and compare the possible conformers and related energies of the neutral forms of GLAC and Ψ-GLAC. In the case of Ψ-GLAC, the additional possibility of tautomerization (structures A–C, Figure 3) was also taken into account, since it was expected to affect the dihedral angle between the pyrimidinone and acridone chromophores, and therefore the conformation was most probably adopted when binding to the catalytic site of RMGP. Detailed crystallographic studies on RMGP:GLAC complexes [25] have revealed that (a) the two chromophores in GLAC extend in a linear-like conformation, where the dihedral angles ψ_1_ (*N*^3^*C*^4^*N*^4^*C*^2^) and ψ_2_ (*C*^4^*N*^4^*C*^2^C^1^, Figure 4) are in the s-cis and s-trans conformation, respectively, and (b) the two chromophores are near coplanar with ψ_2_ of 168.1°. Since a similar ψ_2_ has been observed with other 1-(β-d-glucopyranosyl)-4-arylaminopyrimidin-2-ones bound in the catalytic site of RMGP, this angle is considered the ideal ψ_2_ for binding in the ensuing discussion.

### Computational Studies

Calculations were performed at the DFT level of theory, using an M062X functional with implicit water (PCM/M062X/6-311g**), as implemented in the Gaussian16 software package [56]. Since, in some cases, we will compare fully optimized systems with partially optimized systems (containing locked dihedral angles), we will include in our discussion not only ∆∆G, but also ∆E, an energy term more appropriate to describe non-stationary points. Most energies given below are ∆∆Gs, except when ∆Es are mentioned.

**Figure 5:**
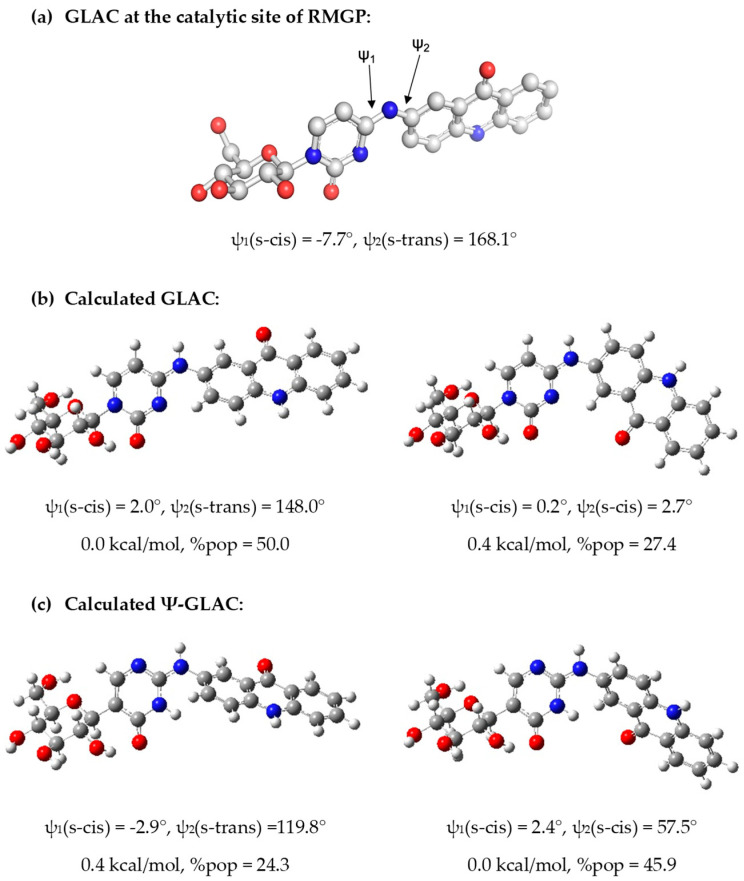
(**a**) Conformation of GLAC within the catalytic site of RMGP. (**b**) Τhe top two lower-energy conformations calculated for GLAC in a polar aqueous environment. (**c**) Τhe top two lower-energy conformations calculated for Ψ-GLAC in a polar aqueous environment.

Initially, we considered the rotation of dihedral angles ψ_1_ and ψ_2_ (Figure 4 and Figure 5b) in GLAC. There are four conformation minima for GLAC (s-cis/s-trans, s-cis/s-cis, s-trans/s-trans and s-trans/s-cis). For neutral GLAC in a polar aqueous environment, all conformer energies were calculated within 1 kcal/mol difference. The most stable, populated at 50.0%, is conformation ψ_1_(s-cis)/ ψ_2_(s-trans), the same conformation observed within the catalytic site of RMGP (Figure 5a) [25]. In this most stable conformer in solution, the dihedral angle ψ_2_ is found at 148.0°. In order for the s-cis/s-trans conformation of GLAC to attain the ψ_1_/ψ_2_ found in the catalytic site (−7.7°/168.1°), it requires an additional ∆E = 0.4 kcal/mol.

A similar number of conformation minima can be found for all three relevant tautomers (**A, B** or **C**, Figure 3) of Ψ-GLAC (Figure 5c). If we compare the energies of the most populated conformers of each tautomer, tautomer **B** is the most stable one (Supplementary Materials Table S1), followed by tautomer **A**. We found experimental evidence that tautomer **B** is the only one observed in the ^1^H NMR spectra of intermediate **15**, in which *H-6* appeared as a singlet at 7.85 ppm, indicating the absence of a vicinal *N^1^-H*. In contrast, the *H-6* in **13** appears as a doublet centered at 7.52 ppm and a similar coupling (5.1 Hz) is exhibited by the vicinal *N^1^-H*. The stability of tautomer **B** in **6** or **15** can be also explained, in chemical terms, by the stability gained by the extensive conjugation of the pyrimidinone ring experienced in tautomer **B**.

For Ψ-GLAC (tautomer **B**), a similar set of four conformers with energies within 0.8 kcal/mol difference, are calculated. In this case, the most stable conformer in solution, populated at 45.9%, is conformation ψ_1_(s-cis)/ψ_2_(s-cis), with the s-cis/s-trans conformer lying 0.4 kcal/mol higher and populated at 24.3%. In order to attain the conformation needed for binding at the catalytic site of RMGP, using the binding mode of GLAC as a reference, Ψ-GLAC’s most stable conformer has to rotate around ψ_2_, overcoming a barrier of 1.9 kcal/mol, to obtain the higher energy s-cis/s-trans conformation. In this latter conformer, the dihedral angle ψ_2_ is found at 119.8°, which is significantly smaller than that of GLAC. This smaller dihedral can be attributed to the presence of the amidic *N^3^*-hydrogen, whose steric hindrance keeps the two chromophores away from coplanarity. If we compare the ∆E of the s-cis/s-trans conformation of Ψ-GLAC with the corresponding ∆E of the conformation with ψ_1_ and ψ_2_ locked at −7.7° and 168.1°, respectively, when bound at the catalytic site of GP for GLAC, we find that an additional amount of energy (∆E = 3.3 kcal/mol) is required.

One may consider the possibility of tautomer **B** transforming to the higher energy tautomer **A** that lacks an *N^3^-*hydrogen and may rotate, similarly to GLAC, in order to obtain a flatter s-cis/s-trans conformation. However, in tautomer **A**, the s-cis/s-trans conformer already lies 2.3 kcal/mol (∆E) higher than the more stable s-cis/s-cis conformer of tautomer **B** and requires an additional ∆E = 0.4 kcal/mol to attain the “optimal” for binding ψ_1_ /ψ_2_. Therefore, we consider only tautomer **B** to be relevant for this discussion.

**Figure 6:**
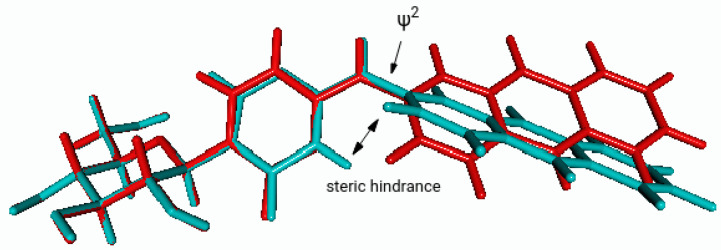
Superimposition of the ψ_1_(s-cis)/ ψ_2_(s-trans) conformers of GLAC (red) and Ψ-GLAC (cyan), with the glucose moieties occupying the same space.

A second salient point to be taken into account, is the structural changes caused by the change in the *C-N* glycosidic bond of GLAC to a *C-C* bond in Ψ-GLAC. It is of interest to consider the angle that the β-anomeric bond directs the chromophores. Since the binding of glucose in the catalytic site of RMGP is tight and very well-defined, superimposition of the glucose moieties in both GLAC and Ψ-GLAC, in their respective ψ_1_(s-cis)/ ψ_2_(s-trans) conformations (Figure 6), provides a clear image of whether the chromophores in both molecules extend towards the direction of the β-channel within the catalytic site [16]. As can be seen in Figure 6, both the sugar and pyrimidinone relative geometries, as well as the β-anomeric bond direction, do not change substantially, going from GLAC to Ψ-GLAC, and the only major change observed is the dihedral angle ψ_2,_ which changes from 148.0° (GLAC) to 119.6° (Ψ-GLAC), due to the presence of *N*^3^-H and its steric interaction with the *C*^3^-H of acridone.

A third salient point concerns the effect of exchange of the 5-*CH* group in GLAC with a nitrogen atom (*N^1^*) in Ψ-GLAC. As pointed out by the DFT calculations, the geometry of both the sugar and pyrimidinone moieties are expected to be very similar in their respective ψ_1_(s-cis)/ ψ_2_(s-trans) conformation (Figure 6). Previous crystallographic studies of the RMGP:GLAC complex [25] reveal that the 5-*CH* group of the pyrimidinone ring is involved in a number of van der Waals non-polar/non-polar and non-polar/polar interactions with the surrounding residues at the catalytic site. More specifically, the *C^5^* carbon atom in GLAC exhibits non-polar/non-polar interactions with the CG2 methyl–carbon atom of Thr 378 (3.8 Å), and CB methyl carbon atom of His 377 (3.8 Å) and a non-polar/polar interaction with the OD2 oxygen atom of Asp 339 (3.8 Å). In Ψ-GLAC, the 5-*CH* group of GLAC is replaced by a nitrogen atom (*N^1^*) and the exchange of the previous non-polar/non-polar with polar/non-polar interactions is not expected to lead to any substantial energy differences. Only the interaction with the OD2 carboxylate oxygen atom of Asp 339 is expected to be repulsive, but rotation of the carboxylate is plausible, as observed in previous RMGP crystal structures. We therefore surmise that the overall change will have only a minor effect in the binding affinity of Ψ-GLAC at the catalytic site, although crystallographic studies of the RMGP:Ψ-GLAC complex are necessary in order to clarify this point.

We propose that the above analysis provides an explanation for the profound 45-fold decrease in Ψ-GLAC’s inhibitory activity towards RMGP when compared with GLAC. Specifically, the calculations showed that GLAC’s most stable and populated s-cis/s-trans conformation in solution is the one required by the catalytic site for binding. On the other hand, Ψ-GLAC’s most stable s-cis/s-cis conformation lies behind a 1.9 kcal/mol barrier in order to obtain the necessary for binding, s-cis/s-trans conformation, and the total cost to obtain the dihedral angles, observed in the catalytic site is ∆E = 3.4 kcal/mol. Pivotal for the observed decrease in activity is the amidic *N^3^*-hydrogen that hinders the two chromophores from assuming near-coplanarity (Figure 6).

It is useful to compare the activity of these new, *C*-glycosyl pyrimidin-4-one derivatives with the structurally related 2-(glucosyl)pyrimidin-4-ones [57,58], recently reported to exhibit no activity towards RMGP. The 4-carbonyl group of the compounds synthesized herein (**4**, **5**, **6**), positioned ortho to the *C*-glycosyl bond, is expected to be pointing in the same direction as was observed for the 2-carbonyl group of GLAC (Figure 2 and Figure 6). In the crystal structure of the RMGP:GLAC complex [25], this carbonyl forms water-mediated interactions with the carboxylates of Glu 88 and Asp 283. We believe that these interactions are crucial for binding, since, in the case of 2-(glucosyl)pyrimidin-4-ones, where this carbonyl group is replaced by a pyrimidine nitrogen, no activity is observed [57,58].

## 4. Materials and Methods

All reagents and solvents were purchased from commercial sources and used without further purification, unless otherwise stated. All reactions were carried out under an argon atmosphere on a magnetic stirrer and monitored by thin-layer chromatography. Compounds were purified by flash chromatography on silica gel 40–60 μm, 60 Å. NMR measurements were performed with a Varian Mercury 200 Nuclear Magnetic Resonance Spectrometer (at 200 MHz for ^1^H and at 50 MHz for ^13^C) and with a Bruker Avance 400 (at 400 MHz for ^1^H and at 101 MHz for ^13^C). Chemical shifts are given in ppm and were referenced on residual solvent peaks, whereas for the spectra of **5** in D_2_O, acetic acid was used as an internal reference. Coupling constants were measured in Hz. High-Resolution Mass Spectrometry experiments were carried out in a Q-TOF Bruker MaXis Impact HR-Mass Spectrometer. 5-Bromo-2-(methylthio)pyrimidin-4-one [37,38], 2,3,4,6-tetra-*O*-benzyl-d-glucono-1,5-lactone (**9**) [46,59,60] and 2-aminoacridin-9(10*H*)-one [55] were synthesized by previously reported procedures.

**5-Bromo-4-*tert*-butoxy-2-methylthiopyrimidine (8).** Freshly distilled phosphorus oxychloride (1.27 mL, 13.6 mmol) was added to 5-bromo-2-(methylthio)pyrimidin-4-one (**7**) (1.00 g, 4.52 mmol), and the mixture was heated to reflux under the exclusion of moisture by a calcium chloride guard tube. After 2 h, the excess chlorinating agent was distilled off under vacuum, using a short vigreux column to avoid co-distillation of the product. The residue was taken up in diethyl ether, washed three times with saturated aqueous sodium bicarbonate solution, and the organic layer was dried over anhydrous sodium sulfate and concentrated under vacuum. The yellow oily residue (Caution: strong sensitizer!) was dissolved in 10 mL of anhydrous THF and cooled in an ice bath, under argon. Potassium *tert*-butoxide (585 mg, 5.20 mmol) was added to the mixture as a 1.45 M solution in anhydrous THF, over 45 min. The mixture was allowed to warm to room temperature and was left stirring for 16 h. The solvent was removed under vacuum and the residue taken up in diethyl ether. The etheric solution was washed once with aqueous sodium hydroxide (1 M) and three times with water. The organic layer was dried over anhydrous sodium sulfate and concentrated. The yellow residue was distilled at 10^−1^ mm Hg by short path distillation, to isolate 1.13 g (90%) of title compound as a low melting, off-yellow solid. **^1^H NMR (200 MHz, CDCl_3_)** δ 8.3 (s, 1H), 2.5 (s, 3H), 1.6 (s, 9H). **^13^C NMR (50 MHz, CDCl_3_)** δ 169.8, 164.1, 158.0, 102.7, 84.0, 28.2, 14.6. **HRMS (ESI/Q-TOF) m/z:** [M + H]^+^Calcd for C9H14BrN2OS^+^ 277.0005; Found 277.0008.

**5-(2,3,4,6-Tetra-*O*-benzyl-d-glucopyranos-1-yl)-4-(*tert*-butoxy)-2-(methylthio)pyrimidine (10).** iPrMgCl·LiCl (1.3 M in anhydrous THF, 6.5 mL, 8.45 mmol) over 30 min was added to a solution of **8** (1.80 g, 6.50 mmol) in 6 mL of anhydrous THF, cooled to −22 °C, over 30 min. The mixture was stirred at the same temperature for 3 h, after which thin layer chromatography showed consumption of the starting material. Over the next 40 min, a solution of **9** (2.69 g, 5.00 mmol) in 5 mL of anhydrous THF was added. The reaction mixture was allowed to slowly warm to room temperature and left to stir overnight. After completion, the reaction was quenched by addition of a saturated aqueous solution of ammonium chloride. The organic solvent was removed under reduced pressure and the residue was taken up in 40 mL of DCM. The layers were separated, and the aqueous portion was diluted with water and further extracted with a further 20 mL portion of DCM. The combined organic extracts were washed once with distilled water, once with saturated sodium chloride solution, and the organic layer was dried over anhydrous sodium sulphate, and the solvent removed by rotary evaporation. The yellow oily residue that was obtained after aqueous workup was triturated with 5% EtOAc in petroleum ether. The white precipitated solid was filtered to collect 2.73 g (74%) of spectroscopically pure **4** as a white solid. **^1^H NMR (400 MHz, Acetone-*d*_6_)** δ 8.57 (s, 1H), 7.39–7.24 (m, 15H), 7.20–7.17 (m, 3H), 7.07–7.01 (m, 2H), 5.52 (s, 1H), 4.92 (d, *J* = 11.0 Hz, 1H), 4.90 (s, 2H), 4.75 (d, *J* = 11.1 Hz, 1H), 4.66 (d, *J* = 11.3 Hz, 1H), 4.59 (d, *J* = 12.0 Hz, 1H), 4.54 (d, *J* = 12.1 Hz, 1H), 4.26 (d, *J* = 11.3 Hz, 1H), 4.24 (d, *J* = 9.3 Hz, 1H), 4.08 (t, *J* = 9.2, 9.2 Hz, 1H), 4.08 (dt, *J* = 9.9, 1.9, 1.9 Hz, 1H), 3.82 (dd, *J* = 10.6, 3.6 Hz, 1H), 3.80 (t, *J* = 9.6, 9.6 Hz, 1H), 3.66 (dd, *J* = 10.8, 1.9 Hz, 1H), 2.52 (s, 3H), 1.58 (s, 9H). **^13^C NMR (101 MHz, Acetone-*d*_6_)** δ 171.8, 166.5, 157.3, 140.1, 139.8, 139.6, 139.1, 129.05 (3C), 129.02 (2C), 128.90, 128.89, 128.7 (2C), 128.59 (2C), 128.55 (2C), 128.49, 128.48, 128.3, 128.21, 128.20, 128.18 (2C), 119.3, 97.54, 97.52, 84.5, 83.6, 81.9, 79.2, 76.0, 75.40, 75.38, 73.84, 72.80, 70.0, 28.8, 14.3. **HRMS (ESI/Q-TOF) m/z:** [M + Na]^+^ Calcd for C_43_H_48_N_2_O_7_SNa^+^ 759.3074; Found 759.3090.

**5-(2,3,4,6-Tetra-*O*-benzyl-β-d-glucopyranosyl)-2-(methylthio)pyrimidin-4(3*H*)-one (11).** A total of 700 mg powdered, activated 4 Å molecular sieves was added to a mixture consisting of **12** (778.9, 1.00 mmol) and triethylsilane (1.6 mL, 1.16 g, 10.0 mmol), dissolved in 11 mL of anhydrous dichloromethane. The flask was flushed with argon and trimethylsilyl triflate (505 μL, 622 mg, 2.80 mmol) was added dropwise. The mixture was left stirring for an additional 1 h. The reaction was quenched by addition of saturated sodium bicarbonate solution, diluted with dichloromethane, filtered and the organic phase was separated, washed with 20 mL of deionized water, dried over anhydrous sodium sulfate and concentrated under reduced pressure. The residue was purified by flash column chromatography (EtOAc: PE 4:6) to afford title compound as a white powder (518 mg, 78%). **^1^H NMR (200 MHz, Acetone-*d*_6_)** δ 7.92 (s, 1H), 7.45–7.07 (m, 20H), 4.89 (d, *J* = 11.2 Hz, 3H), 4.74 (d, *J* = 11.2 Hz, 1H), 4.69 (d, *J* = 11.0 Hz, 1H), 4.63 (d, *J* = 12.3 Hz, 1H), 4.51 (d, *J* = 12.1 Hz, 1H), 4.48 (d, *J* = 11.4 Hz, 1H), 4.44 (d, *J* = 9.5 Hz, 1H), 3.95 (t, *J* = 8.9 Hz, 1H), 3.84–3.52 (m, 5H), 2.54 (s, 3H). **^13^C NMR (50 MHz, Acetone-*d*_6_)** δ 163.0, 162.1, 153.7, 140.0, 139.7 (2C), 139.5, 129.0 (4C), 128.8 (2C), 128.7 (2C), 128.5 (3C), 128.45 (3C), 128.3 (2C), 128.14 (2C), 128.09 (2C), 121.7, 87.9, 82.26, 80.25, 79.2, 75.8, 75.4, 75.3, 75.0, 73.8, 70.1, 13.3. **HRMS (ESI/Q-TOF) m/z:** [M + H]^+^ Calcd for C_39_H_41_N_2_O_6_S^+^ 665.2680; Found 665.2674.

**5-(1-*O*-Acetyl-2,3,4,6-tetra-*O*-benzyl-d-glucopyranos-1-yl)-2-(methylthio)-4-(*tert*-butoxy) pyrimidine (12)**. Sodium hydride (60% suspension in mineral oil, 140 mg, 3.50 mmol) was washed free of mineral oil by repeated mixing with petroleum ether and decantation, and the solid was dried in a stream of argon. To an ice cooled suspension of the hydride in 3 mL of anhydrous THF, was slowly added a solution of **10** (500 mg, 0.679 mmol) in 3 mL of anhydrous THF. After 1 h, acetic anhydride (285 μL, 3.00 mmol) was added dropwise; the mixture was allowed to slowly warm to room temperature and was stirred for 72 h. The reaction was quenched by careful addition of 1 mL of deionized water and the solvent removed under vacuum. The residue was taken up in water and extracted 2 times with 20 mL of diethyl ether. The combined organic extracts were washed twice with distilled water, the organic layer was dried over anhydrous sodium sulfate, and the solvent removed by rotary evaporation. The compound was used directly in the next step without further purification (quantitative crude yield). **^1^H NMR (400 MHz, Acetone-*d*_6_)** δ 8.28 (s, 1H), 7.47–7.08 (m, 20H), 4.90 (d, *J* = 10.9 Hz, 1H), 4.89 (s, 2H), 4.71 (d, *J* = 10.9 Hz, 1H), 4.69 (d, *J* = 11.5 Hz, 1H), 4.63 (d, *J* = 12.1 Hz, 1H), 4.59 (d, *J* = 12.1 Hz, 1H), 4.28 (d, *J* = 11.5 Hz, 1H), 4.12 (t, *J* = 8.9 Hz, 1H), 3.91–3.80 (m, 1H), 3.80–3.73 (m, 1H), 3.70 (dd, *J* = 10.7, 1.4 Hz, 1H), 2.52 (s, 3H), 2.24 (s, 3H), 1.53 (s, 9H). **^13^C NMR (50 MHz, Acetone-*d*_6_)** δ 171.1, 168.0, 165.4, 157.0, 139.6, 139.4, 139.3, 138.9, 129.0 (5C), 128.9(2C), 128.74 (2C), 128.71 (2C), 128.40 (2C), 128.36 (2C), 128.24 (2C), 128.21 (2C), 128.15, 116.1, 101.8, 84.0, 83.7, 83.3, 78.4, 75.9, 75.7, 75.5, 74.1, 73.8, 69.3, 28.6, 21.8, 14.2. **HRMS (ESI/Q-TOF) m/z**: [M + H]^+^ Calcd for C_45_H_51_N_2_O_8_S^+^ 779.3361; Found 779.3366.

**5-(2,3,4,6-Tetra-*O*-benzyl-β-d-glucopyranosyl)pyrimidin-2,4-dione (13).** A total of 30% hydrogen peroxide (68 μL, 0.640 mmol) was added to a solution of **11** (133 mg, 0.200 mmol) in 1.5 mL of glacial acetic acid. The mixture was left stirring at room temperature for 1.5 h and then heated to 70 °C for an additional 2 h. The reaction solution was concentrated under reduced pressure and the residue neutralized by the addition of saturated sodium bicarbonate solution. The mixture was extracted with 20 mL of dichloromethane, the organic phase washed three times with 10 mL of deionized water, separated, dried over anhydrous sodium sulfate and concentrated. The residue was purified by flash column chromatography (Ethyl acetate 50–80% in petroleum ether), to afford the product as 110 mg of white solid (yield 87%). **^1^H NMR (400 MHz, DMSO-*d*_6_)** δ 11.13 (s, 1H), 10.92 (d, *J* = 5.1 Hz, 1H), 7.52 (d, *J* = 5.5 Hz, 1H), 7.38–7.21 (m, 16H), 7.19 (dd, *J* = 7.2, 2.2 Hz, 2H), 7.12 (dd, *J* = 7.2, 2.4 Hz, 2H), 4.82 (s, 2H), 4.75 (d, *J* = 11.0 Hz, 1H), 4.65 (d, *J* = 11.3 Hz, 1H), 4.56 (d, *J* = 12.0 Hz, 1H), 4.53 (d, *J* = 11.8 Hz, 1H), 4.46 (d, *J* = 12.1 Hz, 1H), 4.42 (d, *J* = 11.4 Hz, 1H), 4.25 (d, *J* = 9.8 Hz, 1H), 3.89 (t, *J* = 9.3, 9.3 Hz, 1H), 3.69 (t, *J* = 8.7, 8.7 Hz, 1H), 3.66–3.58 (m, 2H), 3.58–3.47 (m, 2H). **^13^C NMR (101 MHz, DMSO-*d*_6_)** δ 163.6, 150.9, 141.6, 138.7, 138.3, 138.22, 138.20, 128.2 (6C), 128.1 (2C), 127.8 (2C), 127.7 (2C), 127.6, 127.5 (3C), 127.43 (3C), 127.42 (2C), 109.9, 86.2, 80.5, 78.30, 78.29, 78.0, 74.42, 73.99, 73.6, 72.3 (2C), 69.0. **HRMS (ESI/Q-TOF) m/z:** [M + Na]^+^ Calcd for C_38_H_38_N_2_O_7_Na^+^ 657.2571; Found 657.2577.

**5*-*(β-d-Glucopyranosyl)pyrimidin-2,4-dione (4).** Palladium on carbon catalyst was added (10%, 30 mg) to a solution of **13** (100 mg, 0.158 mmol) in 5% dichloromethane in methanol, and the mixture was left to hydrogenate under ambient pressure. After 16 h, the mixture was filtered through celite and concentrated to receive pure **10** in quantitative yield as 43 mg of a white solid. **^1^H NMR (400 MHz, D_2_O)** δ 7.72 (s, 1H), 4.22 (d, *J* = 9.9 Hz, 1H), 3.92–3.77 (m, 1H), 3.71 (dd, *J* = 12.3, 3.0 Hz, 2H), 3.61–3.40 (m, 3H). **^13^C NMR (50 MHz, CD_3_OD)** δ 166.5, 153.7, 143.6, 112.1, 82.2, 79.8, 76.5, 74.2, 71.4, 62.8. **HRMS (ESI/Q-TOF) m/z:** [M + Na]^+^ Calcd for C_10_H_14_N_2_NaO_7_^+^ 297.069; Found 297.0693.

**2-Amino-5-(2,3,4,6-tetra-*O*-benzyl-β-d-glucopyranosyl)pyrimidin-4(3*H*)-one (14)**. A solution of **11** (336 mg, 0.499 mmol) in 5 mL of dichloromethane was stirred vigorously with solid ammonium acetate (5.00 g, 64.9 mmol), the solvents removed and the solid heated to 140 °C, whereupon it melted. The mixture was left stirring at same temperature for 16 h. The residue was taken up in water/ dichloromethane, and the organic phase was separated, washed four times with water, dried over anhydrous sodium sulfate and concentrated. The residue was purified by flash column chromatography (Ethyl acetate 70–100% in petroleum ether), to afford the product as 246 mg of white solid (yield 78%). **^1^H NMR (400 MHz, DMSO-*d*_6_)** δ 10.93 (broad s, 1H), 7.67 (s, 1H), 7.43–7.17 (m, 18H), 7.11 (dd, *J* = 7.2, 2.5 Hz, 2H), 6.57 (s, 2H), 4.84 (d, *J* = 11.4 Hz, 1H), 4.81 (d, *J* = 11.5 Hz, 1H), 4.77 (d, *J* = 11.1 Hz, 1H), 4.61 (d, *J* = 11.2 Hz, 1H), 4.59 (d, *J* = 11.1 Hz, 1H), 4.54 (d, *J* = 12.1 Hz, 1H), 4.48 (d, *J* = 12.2 Hz, 1H), 4.41 (d, *J* = 11.2 Hz, 1H), 4.25 (d, *J* = 9.7 Hz, 1H), 3.95 (t, *J* = 9.3, 9.3 Hz, 1H), 3.70 (t, *J* = 8.6, 8.6 Hz, 1H), 3.67 (dd, *J* = 11.1, 2.0 Hz, 1H), 3.63 (dd, *J* = 11.1, 4.2 Hz, 1H), 3.54 (q, *J* = 9.2, 9.2, 8.8 Hz, 1H), 3.55–3.47 (m, 1H). **^13^C NMR (101 MHz, DMSO-*d*_6_)** δ 161.9, 155.9, 154.9, 140.9, 138.7, 138.4, 138.32, 138.31, 127.92 (5C), 127.88 (2C), 127.7 (2C), 127.41 (2C), 127.39 (2C), 127.18 (4C), 127.1, 127.02, 126.98, 112.1, 86.1, 78.4, 78.3, 74.7, 74.0, 73.6, 73.1, 72.4, 69.4. **HRMS (ESI/Q-TOF) m/z:** [M + Na]^+^ Calcd for C_38_H_39_N_3_O_6_Na^+^ 656.2731; Found 656.2749.

**2-Amino-5-(β-d-glucopyranosyl)pyrimidin-4(3*H*)-one (5).** To a solution of **14** (100 mg, 0.158 mmol) in 5% dichloromethane in methanol, 10% Pd/C catalyst (50 mg) was added and the mixture was left to hydrogenate under ambient pressure. After 24 h, the hydrogen was purged and was replaced by an argon atmosphere, then palladium hydroxide on carbon (20%, 50 mg) was added and the mixture was further hydrogenated for 96 h, after which the mixture was filtered through celite and concentrated. The residue was chromatographed (methanol:water:acetonitrile: ethyl acetate, 1.5:1.5:1.5:5.5) to yield 28 mg (65%) of the title compound as a white solid. **^1^H NMR (200 MHz, D_2_O, 100 °C)** δ 7.7 (s, 1H), 4.2 (d, 1H), 3.9–3.8 (m, 1H), 3.7 (dd, 2H), 3.6–3.4 (m, 3H). **^13^C NMR (101 MHz, D_2_O)** δ 167.9, 156.5, 151.7, 111.8, 80.4, 78.1, 75.8, 71.9, 70.2, 61.4. **HRMS (ESI/Q-TOF) m/z:** [M + Η]^+^ Calcd for C_10_H_15_N_3_O_6_^+^ 274.1034; Found 274.1039.

**2-[(Acridin-9(10*H*)-on-2-yl)amino]-5-(2,3,4,6-tetra-*O*-benzyl-β-d-glucopyranosyl)pyrimidin-4(3*H*)-one (15)**. A suspension of **11** (200 mg, 0.300 mmol) and 4-aminoacridone (79 mg, 0.375 mmol) in 2 mL of pivalic acid were heated to 120 °C for 16 h until the reaction was complete. The volatiles were removed by distillation under high vacuum. The residue was purified by repeated flash column chromatography (first with 80% EtOAc in petroleum ether, then 3% methanol in DCM), to receive 151 mg (61%) of **15 as** a bright yellow powder. **^1^H NMR (400 MHz, DMSO-*d*_6_)** δ 11.78 (s, 1H), 9.15 (s, 1H), 8.42 (d, *J* = 2.5 Hz, 1H), 8.24 (dd, *J* = 8.2, 1.6 Hz, 1H), 7.93 (dd, *J* = 8.9, 2.6 Hz, 1H), 7.85 (s, 1H), 7.71 (ddd, *J* = 8.5, 6.8, 1.6 Hz, 1H), 7.56 (t, *J* = 9.0 Hz, 2H), 7.38–7.15 (m, 18H), 7.14–7.08 (m, 2H), 4.82 (s, 2H), 4.76 (d, *J* = 10.9 Hz, 1H), 4.65 (d, *J* = 11.3 Hz, 1H), 4.57 (d, *J* = 11.3 Hz, 1H), 4.55 (d, *J* = 11.5 Hz, 1H), 4.47 (d, *J* = 12.0 Hz, 1H), 4.42 (d, *J* = 11.4 Hz, 1H), 4.32 (d, *J* = 9.6 Hz, 1H), 3.97 (t, *J* = 9.6 Hz, 1H), 3.71 (t, *J* = 8.4 Hz, 1H), 3.68–3.48 (m, 4H). **^13^C NMR (101 MHz, DMSO-*d_6_*)** δ 176.3, 163.1 (HMBC), 140.7, 138.7, 138.4, 138.3 (2C), 138.0, 137.9, 137.4, 133.3, 131.9, 128.2 (7C), 128.0 (2C), 127.8 (2C), 127.7 (2C), 127.5 (2C), 127.4 (4C), 127.3, 126.0, 120.9, 120.6, 120.0, 118.0, 117.3, 116.3, 114.8, 97.2, 86.3, 80.5, 78.4, 78.2, 74.5, 74.0, 73.6, 72.4 (2C), 69.1. **HRMS (ESI/Q-TOF) m/z:** [M + H]^+^ Calcd for C_51_H_47_N_4_O_7_^+^ 827.3439; Found 827.3442.

**5-(2,3,4,6-Tetra-*O*-acetyl-β-d-glucopyranosyl)-2-(methylthio)pyrimidin-4(3*H*)-one (16)**. Acetic anhydride (15 mL, 1.65 mol) was added to a solution of **11** (674 mg, 1.00 mmol) in 5 mL of dichloromethane and the mixture was cooled in an ice bath under argon. After 10 min, trimethylsilyl triflate (1.45 mL, 8.00 mmol) was added dropwise and the mixture was left stirring at room temperature. After 24 h the dark reaction mixture was cooled in an ice bath and saturated aqueous sodium bicarbonate added slowly. After stirring for 30 min, the mixture was concentrated, the residue taken up in ethyl acetate and washed three times with saturated aqueous sodium bicarbonate, once with water and once with saturated aqueous sodium chloride. The organic phase was dried over anhydrous sodium sulfate, concentrated and subjected to column chromatography (dichloromethane: ethyl acetate: petroleum ether, 20:50:30) to yield 360 mg (76%) of **16** as a white solid. **^1^H NMR (400 MHz, CDCl_3_)** δ 12.39 (s, 1H), 7.97 (s, 1H), 5.34 (t, *J* = 9.2 Hz, 1H), 5.27 (t, *J* = 9.5 Hz, 1H), 5.16 (t, *J* = 9.6 Hz, 1H), 4.70 (d, *J* = 9.7 Hz, 1H), 4.23 (dd, *J* = 12.4, 4.8 Hz, 1H), 4.13 (dd, *J* = 12.4, 2.2 Hz, 1H), 3.81 (ddd, *J* = 10.2, 4.9, 2.1 Hz, 1H), 2.58 (s, 3H), 2.06 (s, 3H), 2.04 (s, 3H), 2.00 (s, 3H), 1.91 (s, 3H). **^13^C NMR (101 MHz, CDCl_3_)** δ 170.8, 170.4, 169.7 (2C), 163.1, 163.0, 153.8, 118.5, 76.3, 74.4, 72.5, 71.6, 68.7, 62.4, 20.9, 20.8, 20.7 (2C), 13.4. **HRMS (ESI/Q-TOF) m/z:** [M + Η]^+^ Calcd for C_19_H_24_N_2_O_10_S^+^ 473.1224; Found 473.1212.

**2-[(Acridin-9(10*H*)-on-2-yl)amino]-5-(β-d-glucopyranosyl)pyrimidin-4(3*H*)-one (6)**. A suspension of **16** (100 mg, 0.212 mmol) and 2-aminoacridin-9(10*H*)-one (60 mg, 0.286 mmol) in 2 mL of pivalic acid was heated to 140 °C for 4 h until complete consumption of **16**. Pivalic acid was removed by distillation under high vacuum. A total of 5 mL of 7M ammonia in methanol was added to the residue and the solution was left to stir overnight. On the next day, solvents were removed under vacuum and the residue repeatedly triturated 10 times with ethyl acetate and five with methanol and collected by centrifugation, to obtain 69 mg of title compound as a bright yellow powder. **^1^H NMR (400 MHz, DMSO-*d*_6,_ D_2_O)** δ 8.42 (s, 1H), 8.21 (d, *J* = 8.2 Hz, 1H), 7.86 (d, *J* = 9.0 Hz, 1H), 7.72 (t, *J* = 7.6 Hz, 2H), 7.53 (t, *J* = 7.5 Hz, 2H), 7.26 (t, *J* = 7.6 Hz, 1H), 4.08 (d, *J* = 9.8 Hz, 1H), 3.64 (d, *J* = 11.7 Hz, 1H), 3.49 (t, *J* = 9.2 Hz, 1H), 3.42 (dd, *J* = 12.0, 4.4 Hz, 1H), 3.24 (t, *J* = 8.2 Hz, 1H), 3.20–3.12 (m, 2H). **^13^C NMR (101 MHz, DMSO-*d*_6,_ D_2_O)** δ 177.7, 164.1, 159.9, 150.1, 140.9, 137.6, 134.1, 132.6, 128.7, 126.4, 121.8, 120.7, 120.1, 118.4, 117.3, 116.4, 108.6, 81.2, 78.6, 75.1, 72.4, 70.4, 61.6. **HRMS (ESI/Q-TOF) m/z:** [M + Na]^+^ Calcd for C_23_H_22_N_4_O_7_Na^+^ 489.1381; Found 489.1385.

RMGP was isolated from rabbit skeletal muscle and its potency was assayed according to previously established protocols [54,61]. More specifically, kinetic studies were performed in the direction of glycogen synthesis at 30 °C, pH 6.8 in the presence of 5 μg/mL enzyme, 2 mM glucose-1-phosphate, 1 mM AMP, 1% glycogen, 1% DMSO and various concentrations of compounds **4**, **5** and **6** ranging from 0.5 to 500 μM (Supplementary Materials Table S3).

## 5. Conclusions

We have successfully developed a synthetic methodology involving a pyrimidone addition to d-gluconolactone, coupled with acetate reduction with alkylsilanes and have utilized a methythio handle to synthesize *C*-glycosyl 2-derivatized-5-β-d-glucopyranosyl-pyrimidin-4-ones. We showcased the possibility of 2*-*derivatization by arylamines, generalizing the synthetic scheme. Kinetic studies, coupled with DFT conformational analysis, allowed for the elucidation of the factors affecting the binding of the new inhibitors to the catalytic site of GP. The crystal structure of the RMGP: Ψ-GLAC complex will reveal more salient differences in the interactions of the two inhibitors within the catalytic site of RMGP and are in progress. Following our theoretical conformational analysis, the design and synthesis of this new family of metabolically stable inhibitors may be further improved by derivatives of Ψ-GLAC that are “locked” in the A tautomeric form. This will be the subject of future studies.

## Supplementary Materials

^1^H and ^13^C NMR spectra of new compounds (Figures S1–S22), results of DFT calculations (Tables S1 and S2) and kinetic study results for compounds **4**, **5**, **6** (Table S3).
