# Supplementary material for: Synthesis, Kinetic and Conformational Studies of 2-Substituted-5-(β-d-glucopyranosyl)-pyrimidin-4-ones as Potential Inhibitors of Glycogen Phosphorylase
Source: Molecules. 2020 Nov 22;25(22):5463. doi: 10.3390/molecules25225463 (PMC7700572; doi:10.3390/molecules25225463)
Supplement: Supplementary file 1 [file molecules-25-05463-s001.pdf]

Synthesis, Kinetic and Conformational Studies of 2-Substituted-5-(β-D-glucopyranosyl)-pyrimidin-4-ones as Potential Inhibitors of Glycogen Phosphorylase

Konstantinos F. Mavreas[1], Dionysios D. Neofytos[2], Evangelia D. Chrysina[2,*] , Alessandro Venturini[3,*] and Thanasis Gimisis[1,*]

1) Laboratory of Organic Chemistry, Department of Chemistry, National and Kapodistrian University of Athens, 10571, Athens, Greece.e-mail: gimisis@chem.uoa.gr.
2) Institute of Chemical Biology, National Hellenic Research Foundation, Athens (Greece). e-mail: echrysina@eie.gr
3) Istituto ISOF, Consiglio Nazionale delle Ricerche. Bologna (Italy). e-mail: alessandro.venturini@isof.cnr.it

Supplementary Materials

Contents

Figures S1-S22. NMR spectra of compounds **4-6, 8, 10, 12-17**

Table S1-2. DFT conformational analysis results

Table S3: Kinetic studies for the calculation of the % inhibition of GPMM activity in the presence of compounds **4**, **5**, **6**

Figure S1. ^{1}H NMR spectrum of compound **8** (200 MHz, $CDCl_3$).

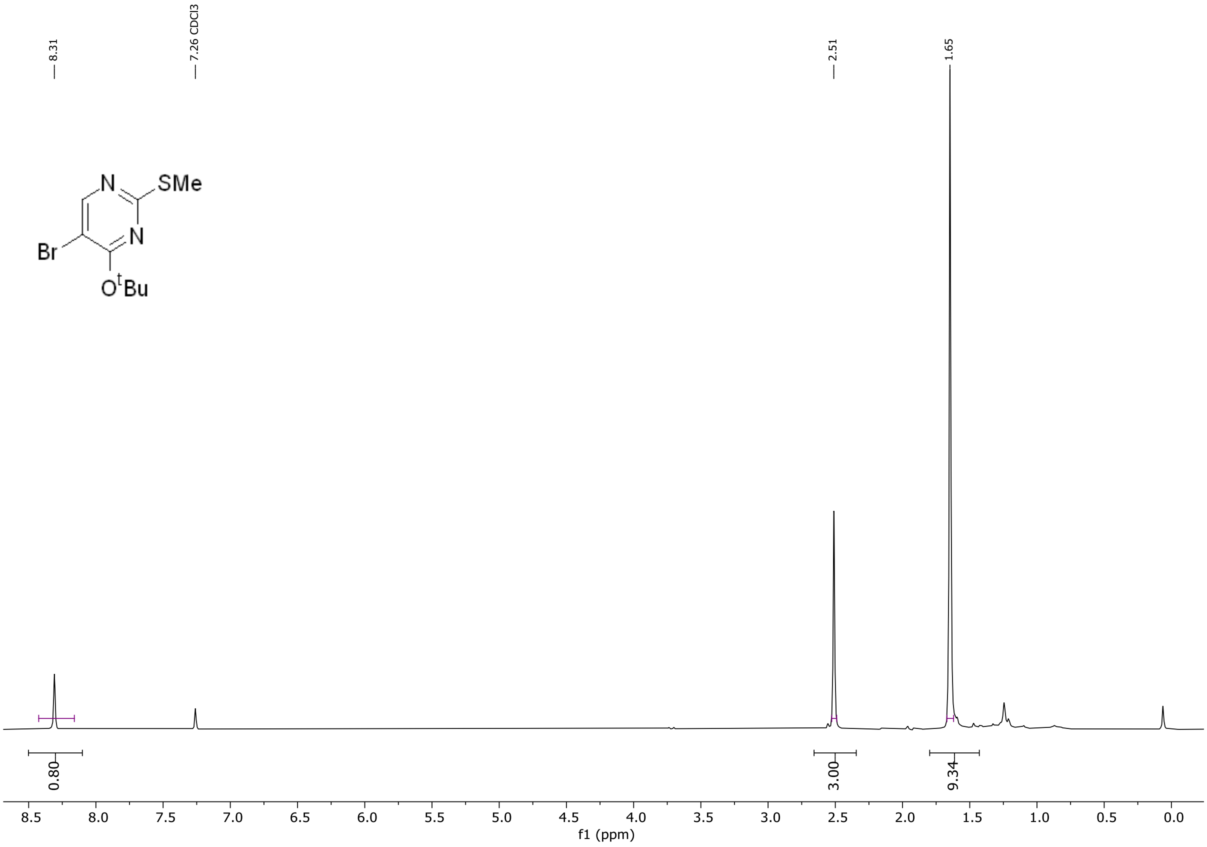

Figure S2. ^{13}C NMR spectrum of compound **8** (50 MHz, $CDCl_3$).

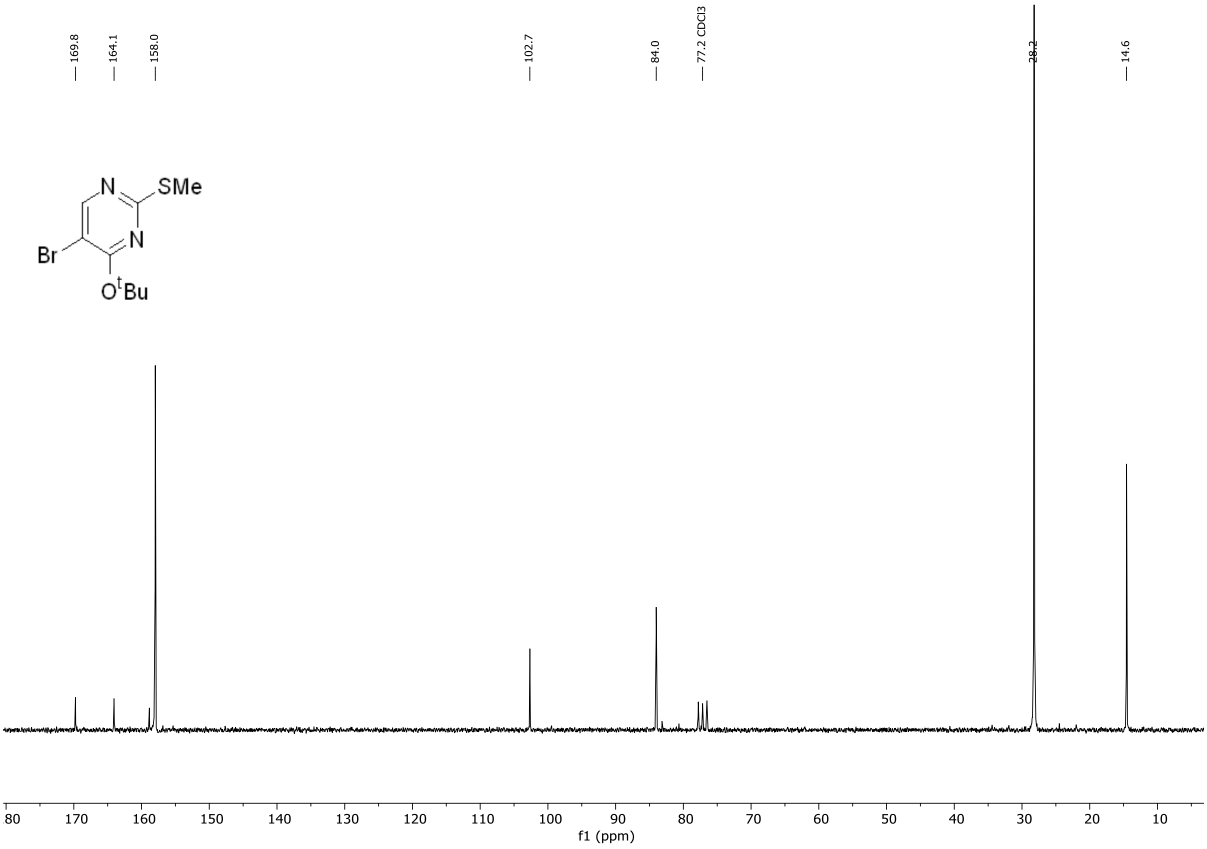

Figure S3. ^{1}H NMR spectrum of compound **10** (400 MHz, Acetone-d$_6$).

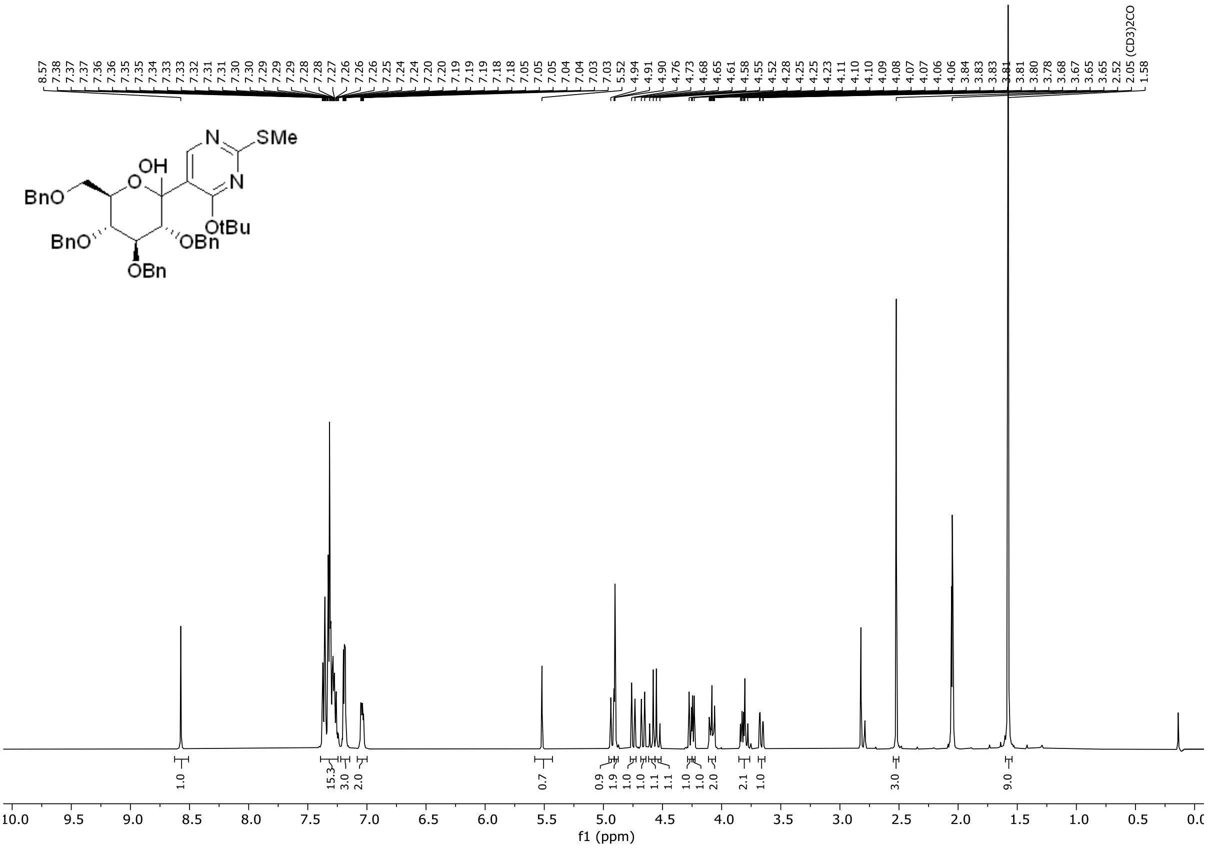

Figure S4. ^{13}C NMR spectrum of compound **10** (50 MHz, Acetone-d$_6$).

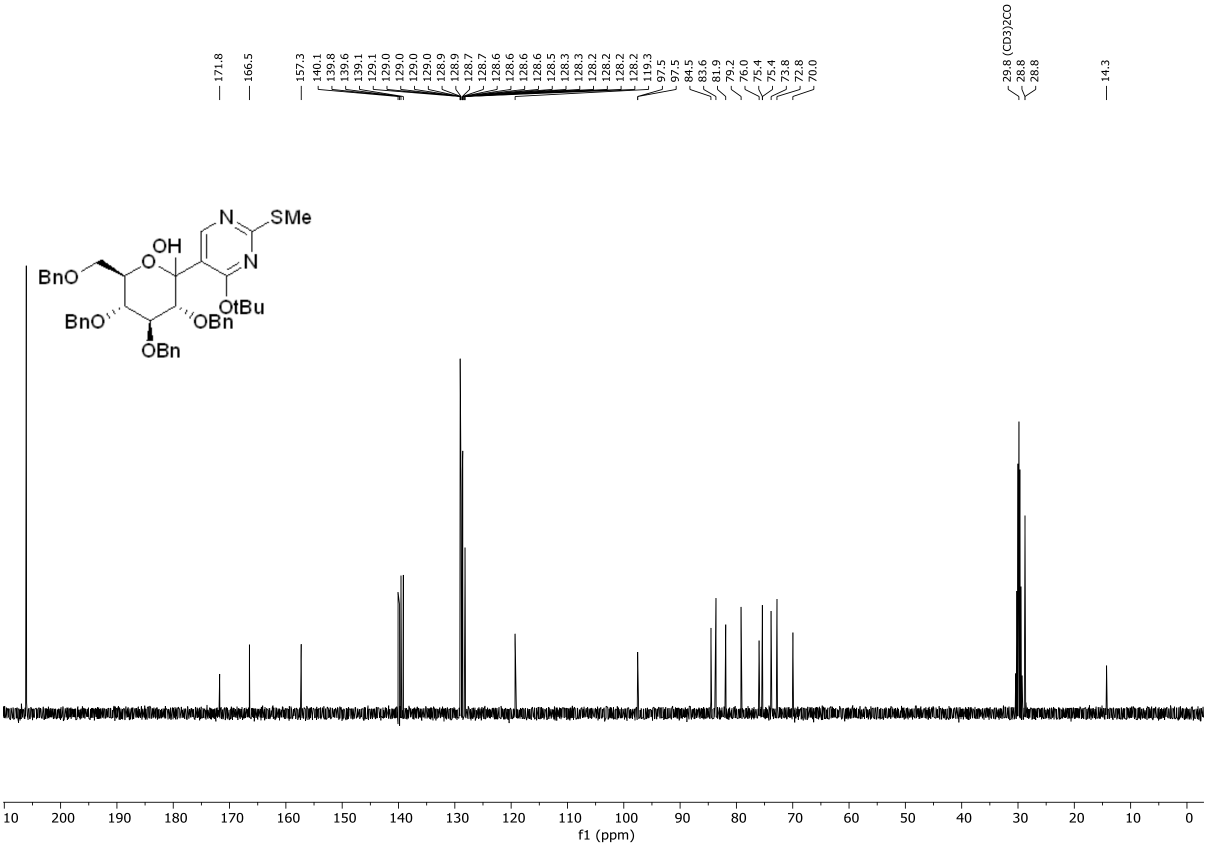

Figure S5. Crude ^{1}H NMR spectrum of compound **12** (400 MHz, Acetone-d6).

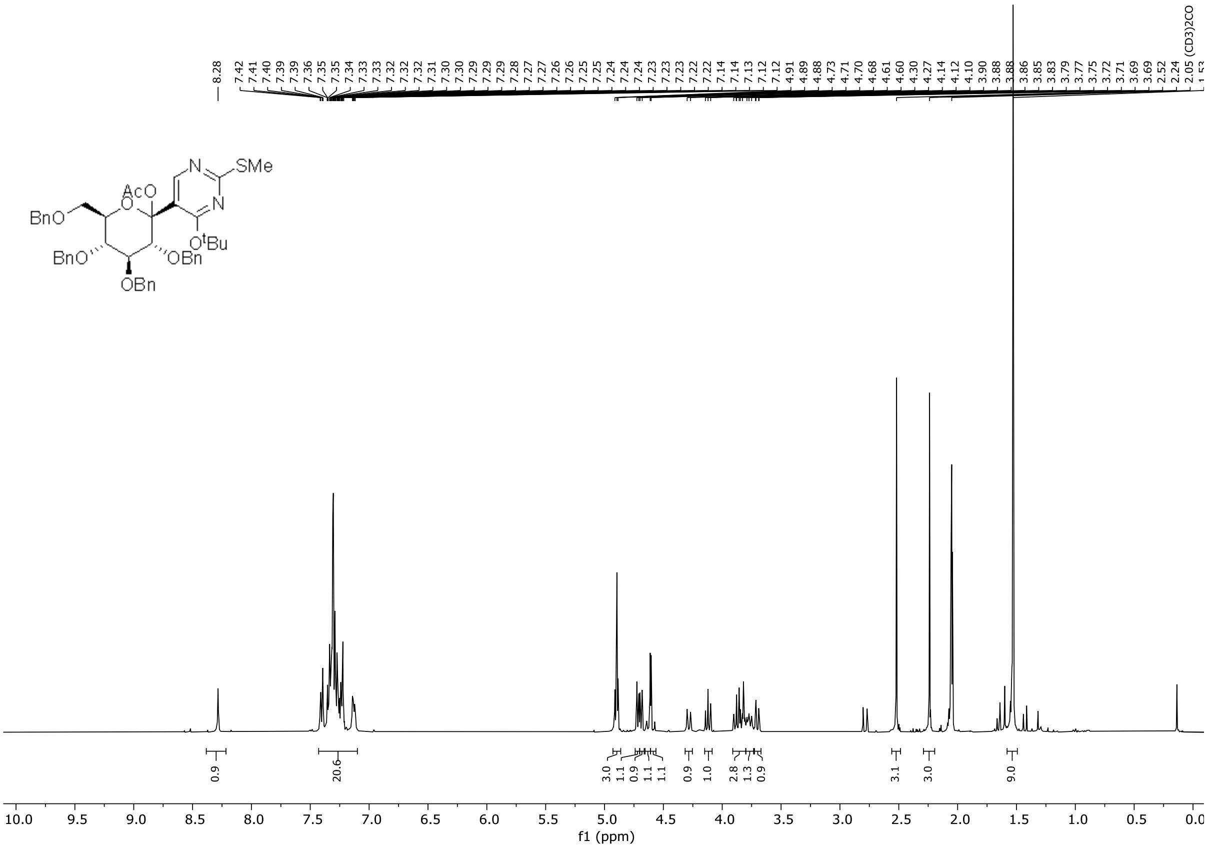

Figure S6. Crude ^{13}C NMR spectrum of compound **12** (50 MHz, Acetone-d6).

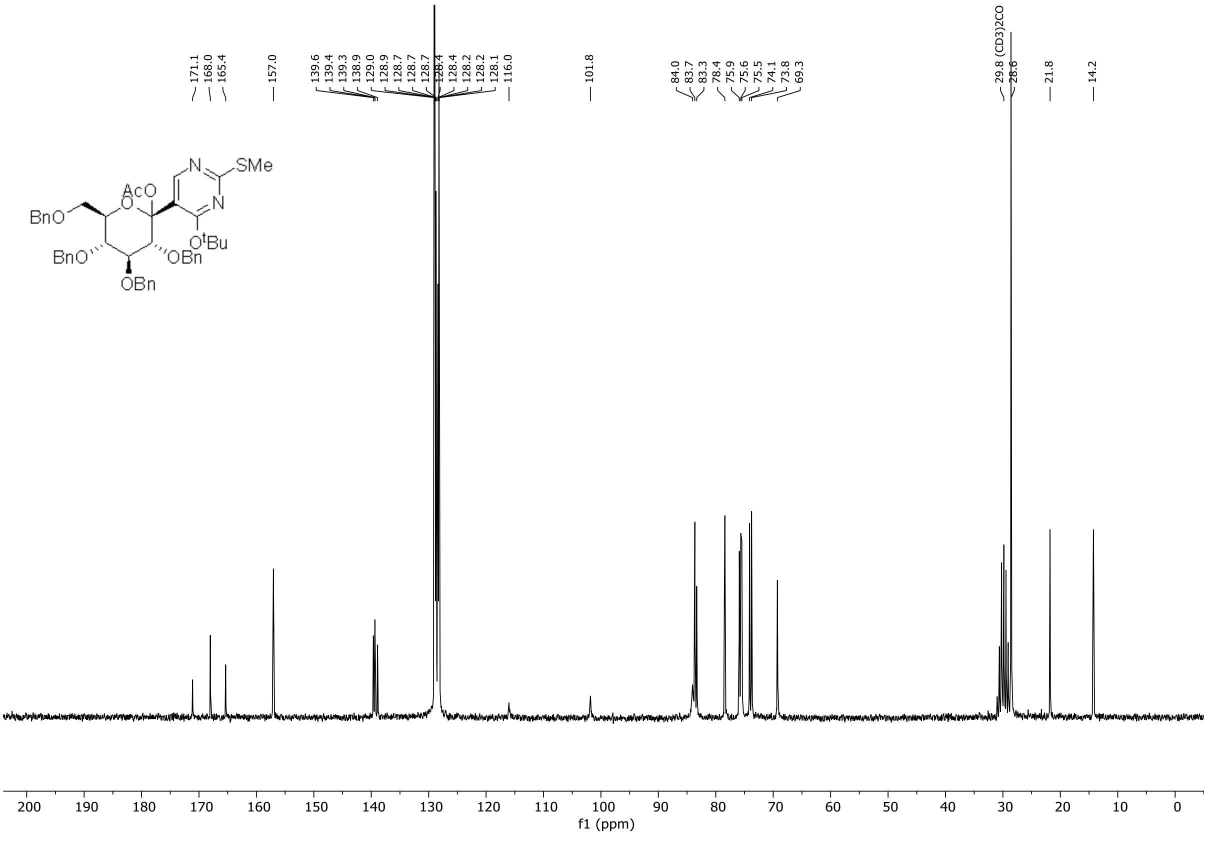

Figure S7. ^{1}H NMR spectrum of compound **11** (200 MHz, Acetone-d_6).

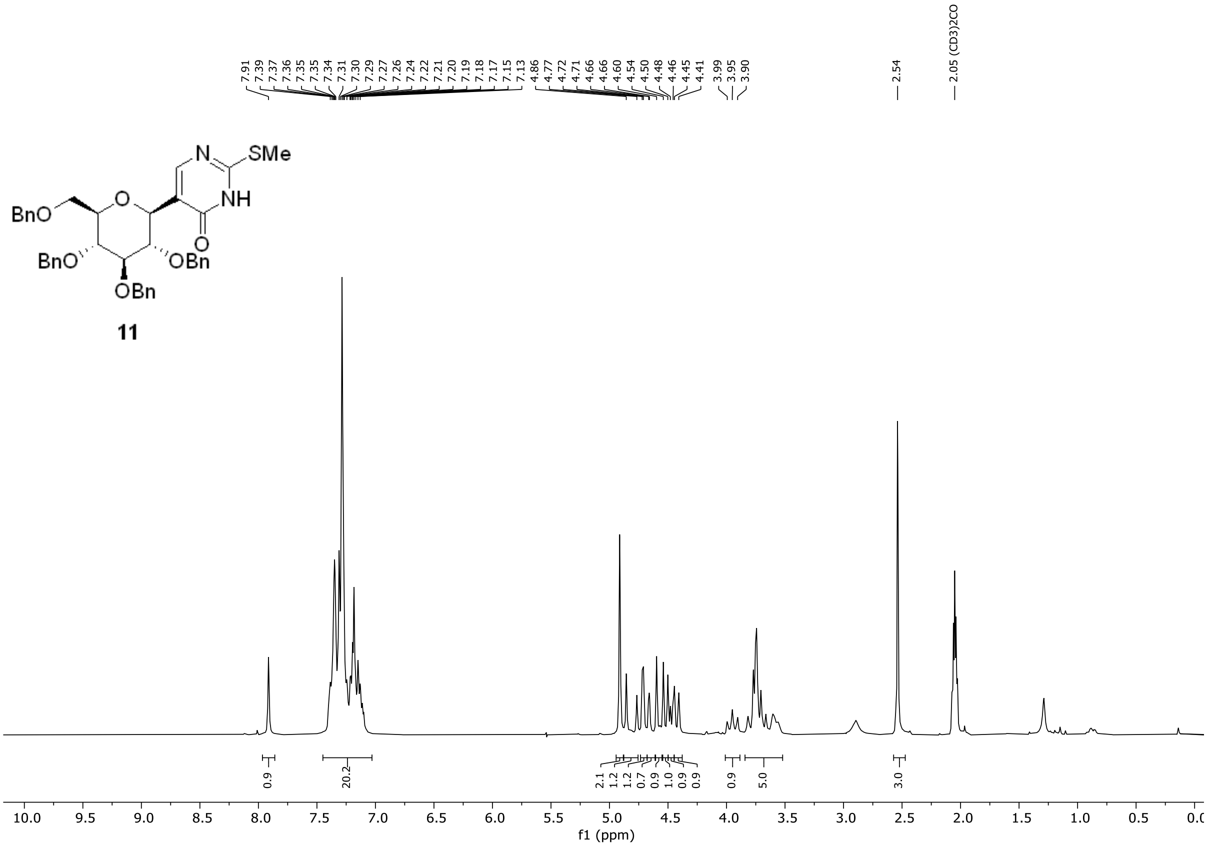

Figure S8. ^{13}C NMR spectrum of compound **11** (50 MHz, Acetone-d_6).

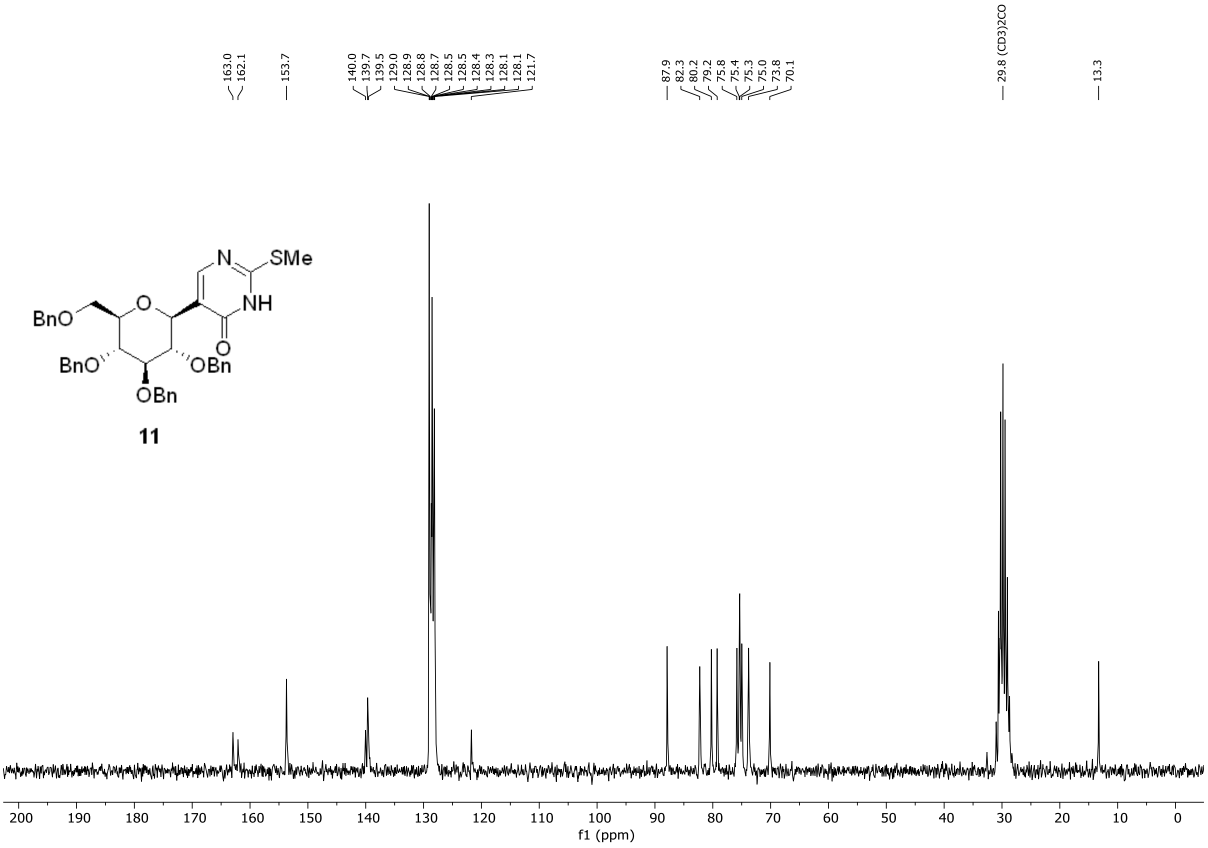

Figure S9. ^{1}H NMR spectrum of compound **13** (400 MHz, DMSO-d$_6$).

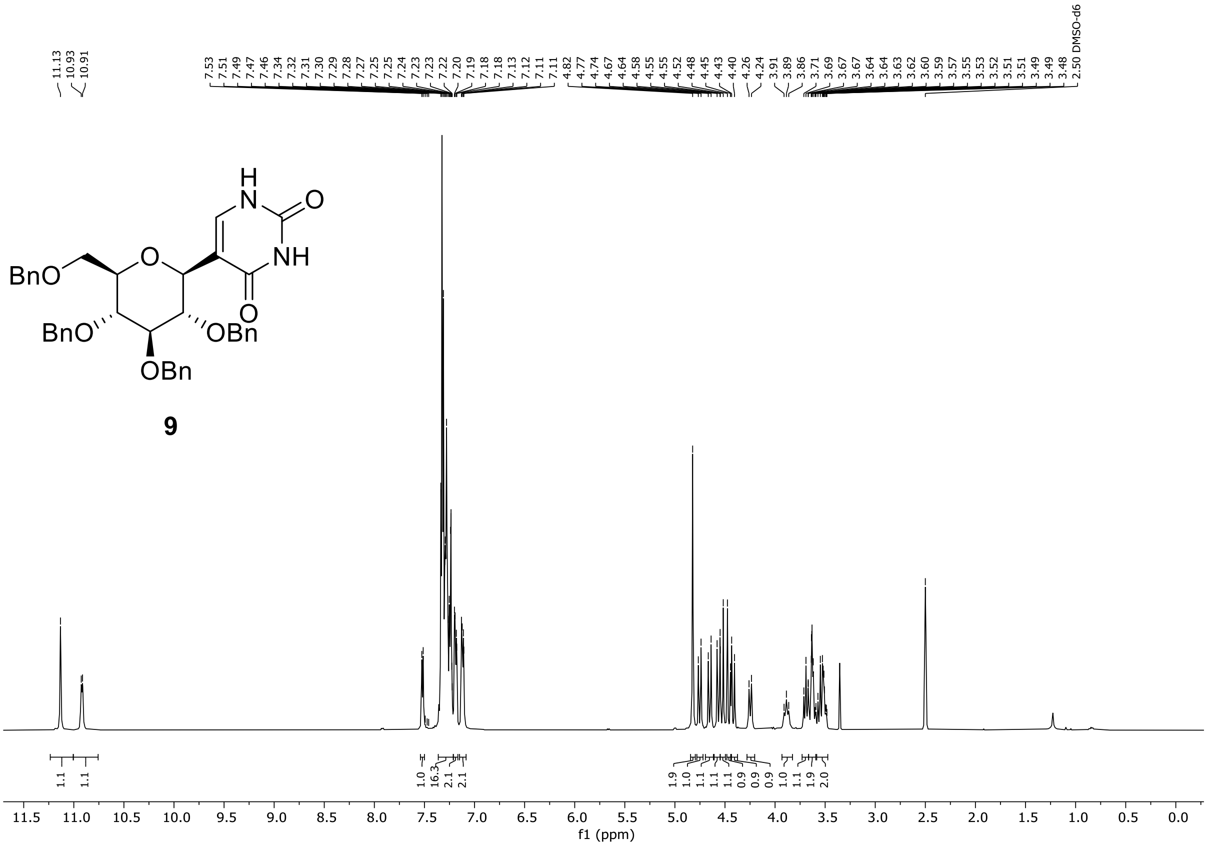

Figure S10. ^{13}C NMR spectrum of compound **13** (100 MHz, DMSO-d$_6$).

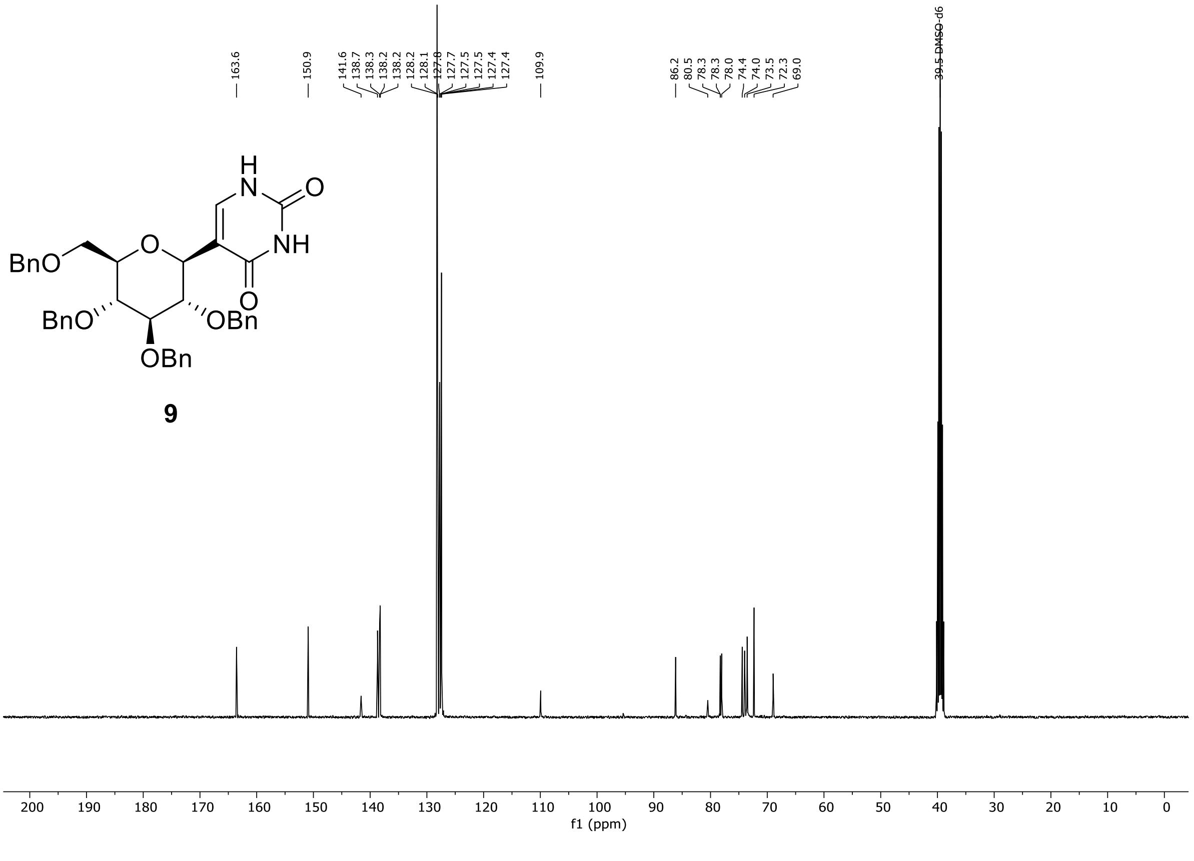

Figure S11. ^{1}H NMR spectrum of compound **4** (400 MHz, D_2O).

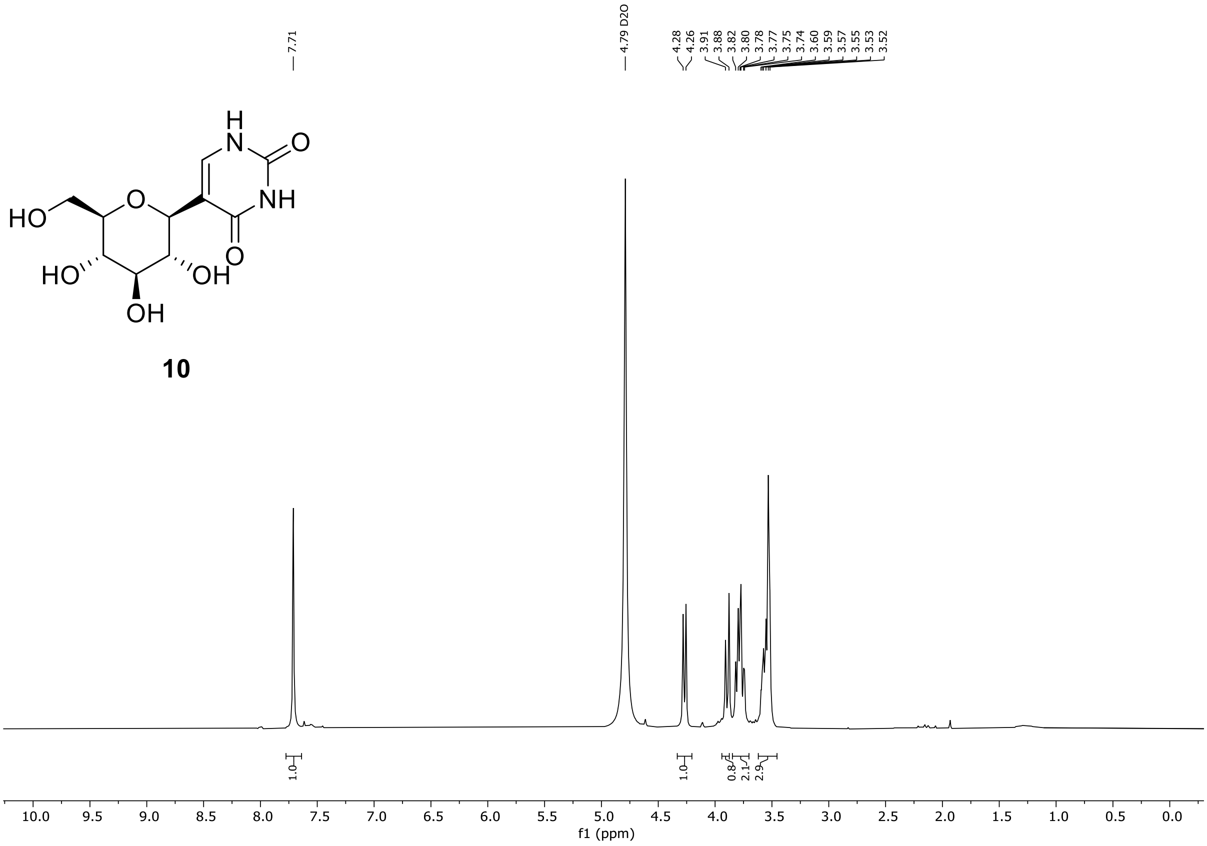

Figure S12. ^{13}C NMR spectrum of compound **4** (50 MHz, CD_3OD).

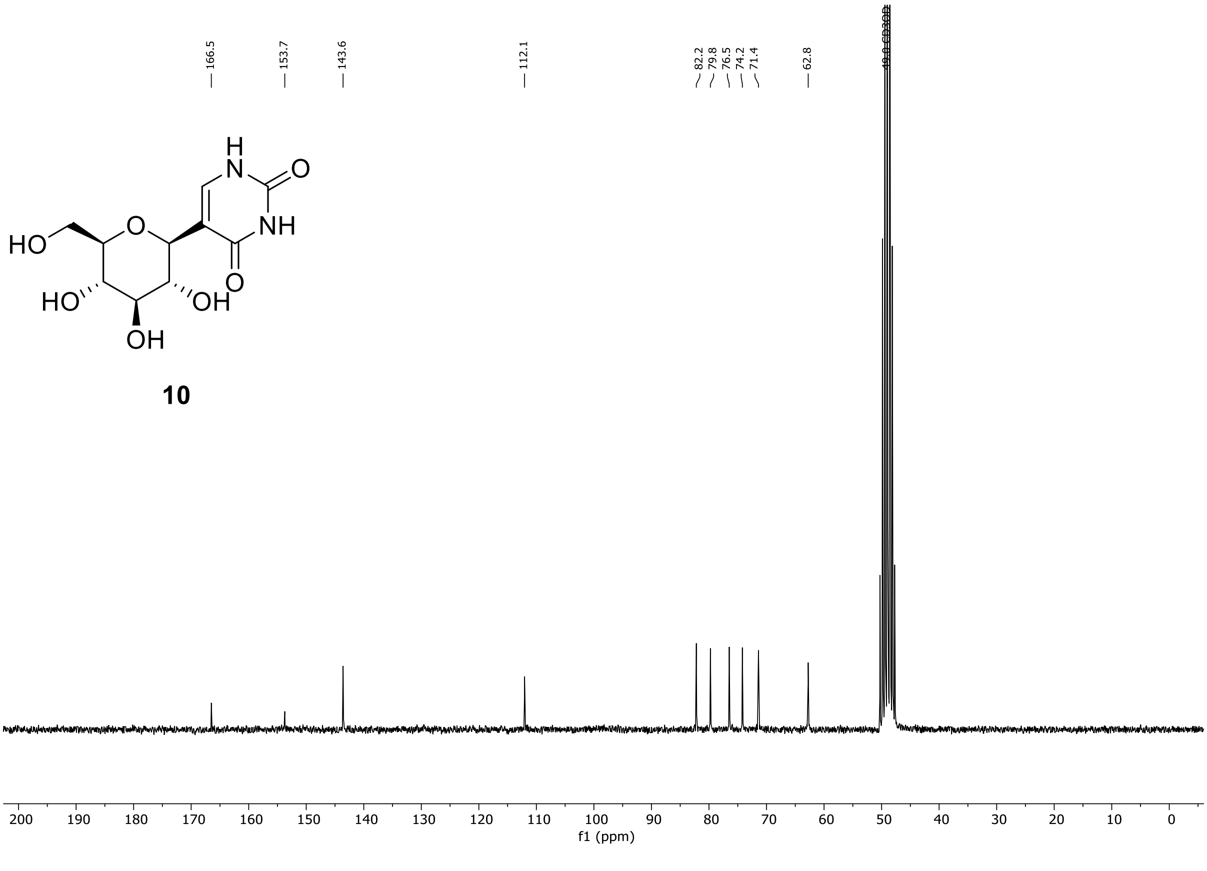

Figure S13. ^{1}H NMR spectrum of compound **14** (400 MHz, DMSO-d_6).

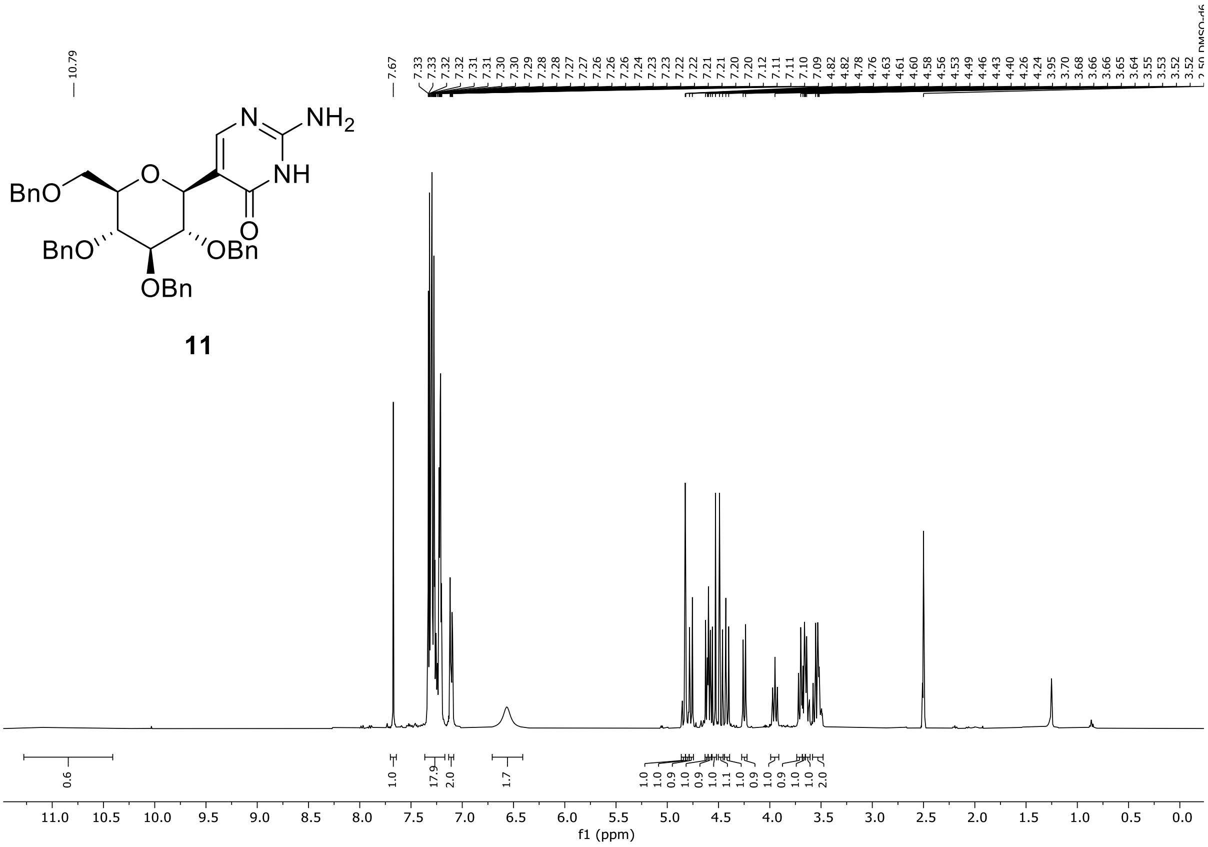

Figure S14. ^{13}C NMR spectrum of compound **14** (100 MHz, DMSO-d_6).

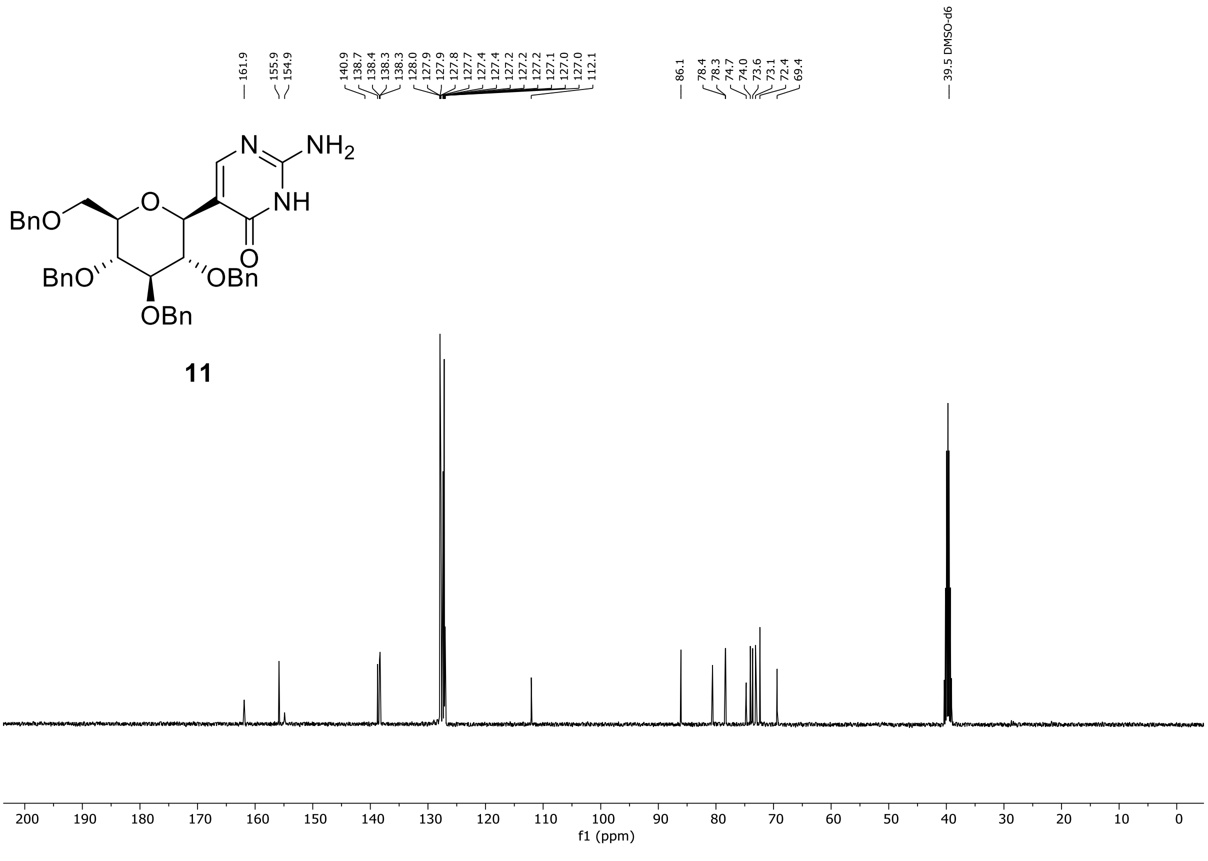

Figure S15 . ^{1}H NMR spectrum of compound **5** (400 MHz, D_2O, 60 °C).

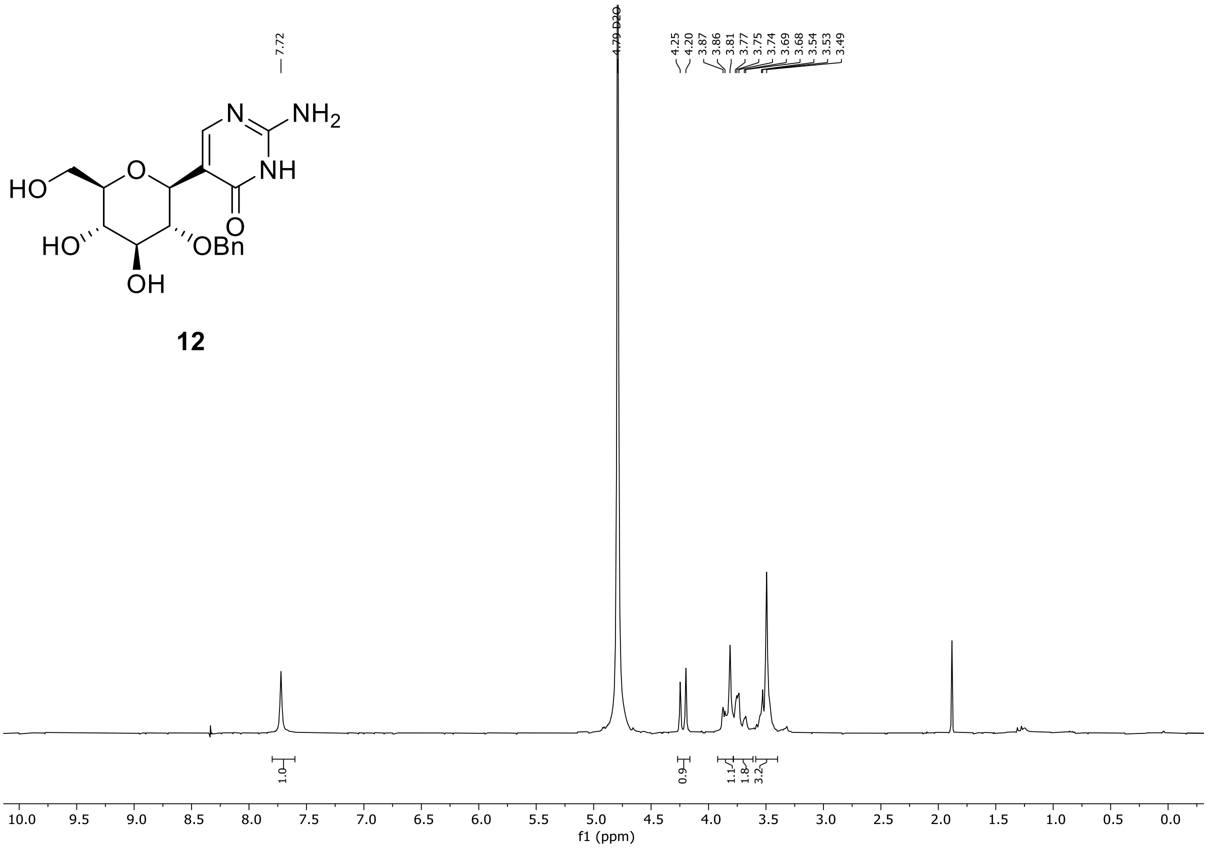

Figure S16. ^{13}C NMR spectrum of compound **5** (100 MHz, D_2O, 60 °C).

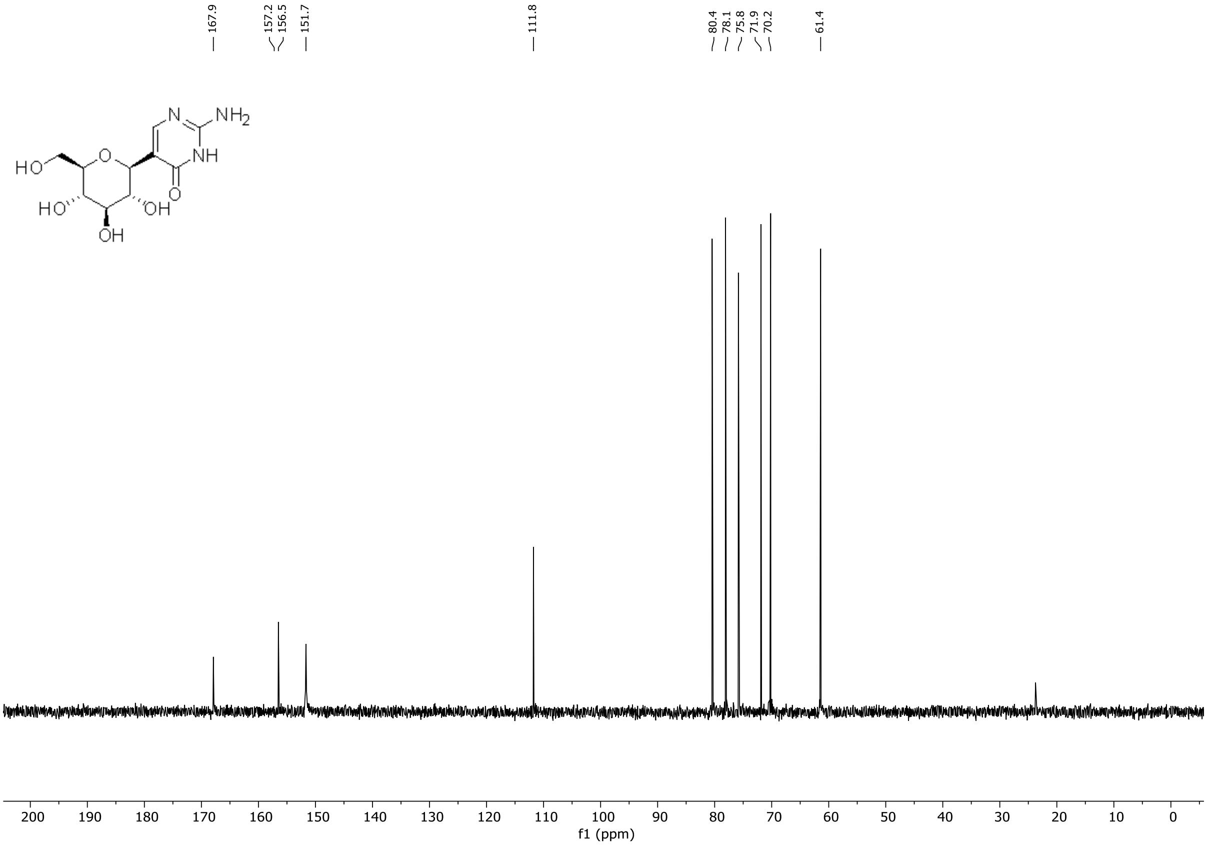

Figure S17 . ^{1}H NMR spectrum of compound **15** (400 MHz, DMSO-d6).

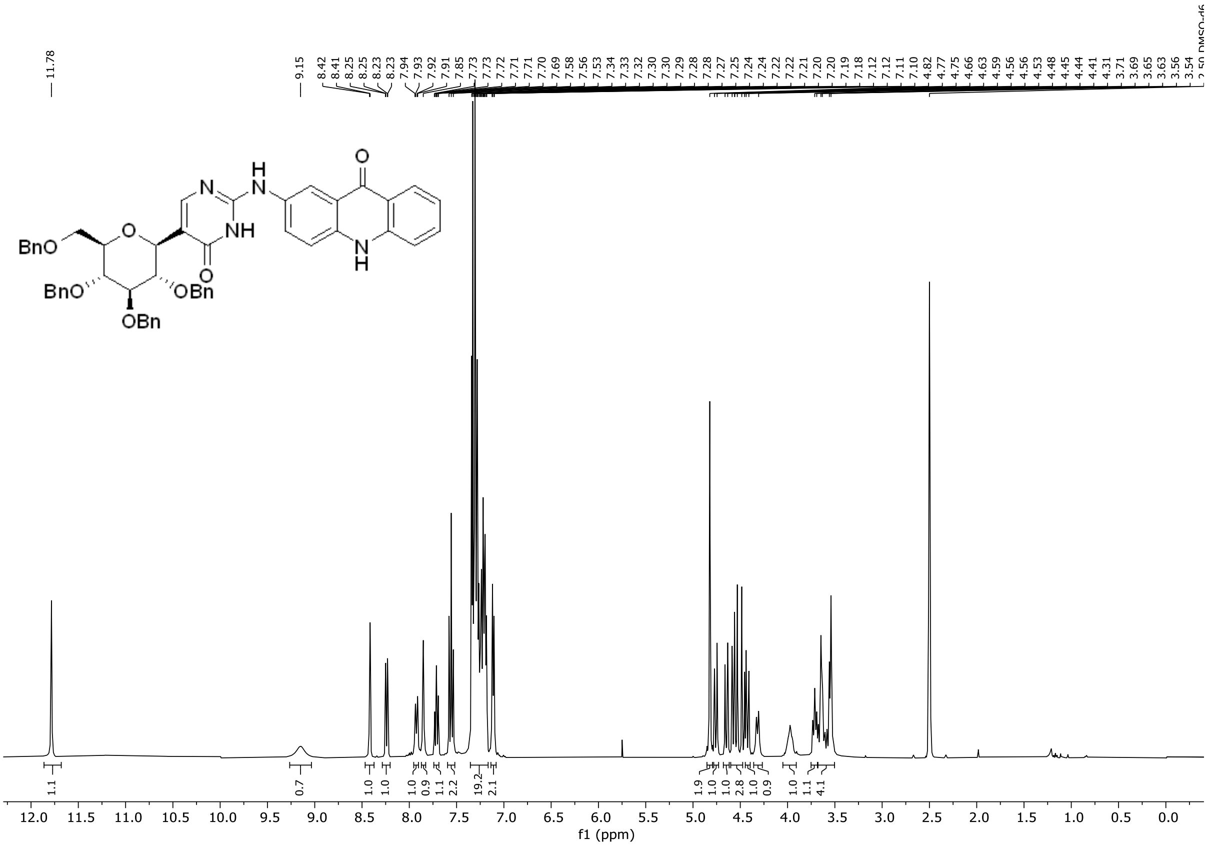

Figure S18. ^{13}C NMR spectrum of compound **15** (50 MHz, DMSO-d6).

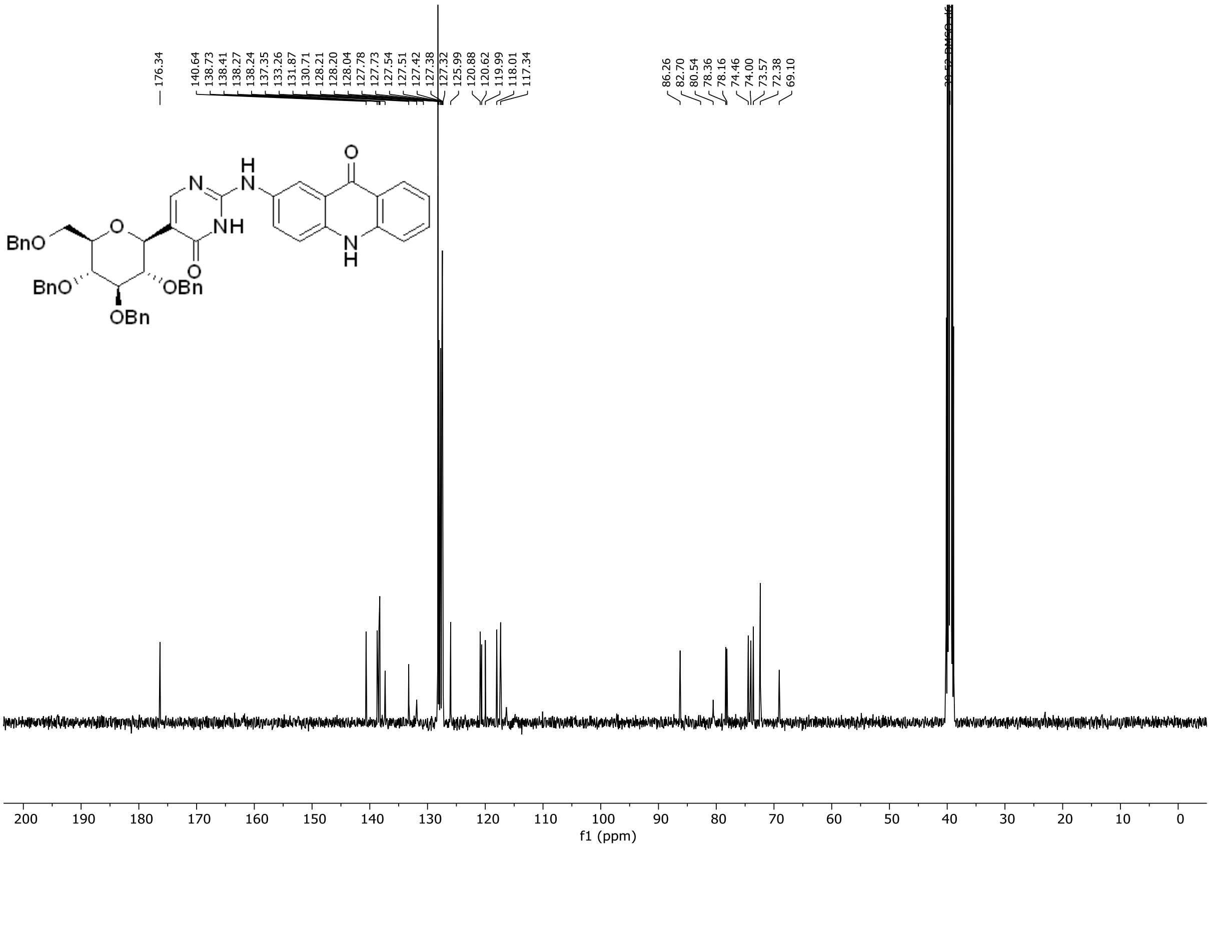

Figure S19 . ^{1}H NMR spectrum of compound **16** (400 MHz, $CDCl_3$).

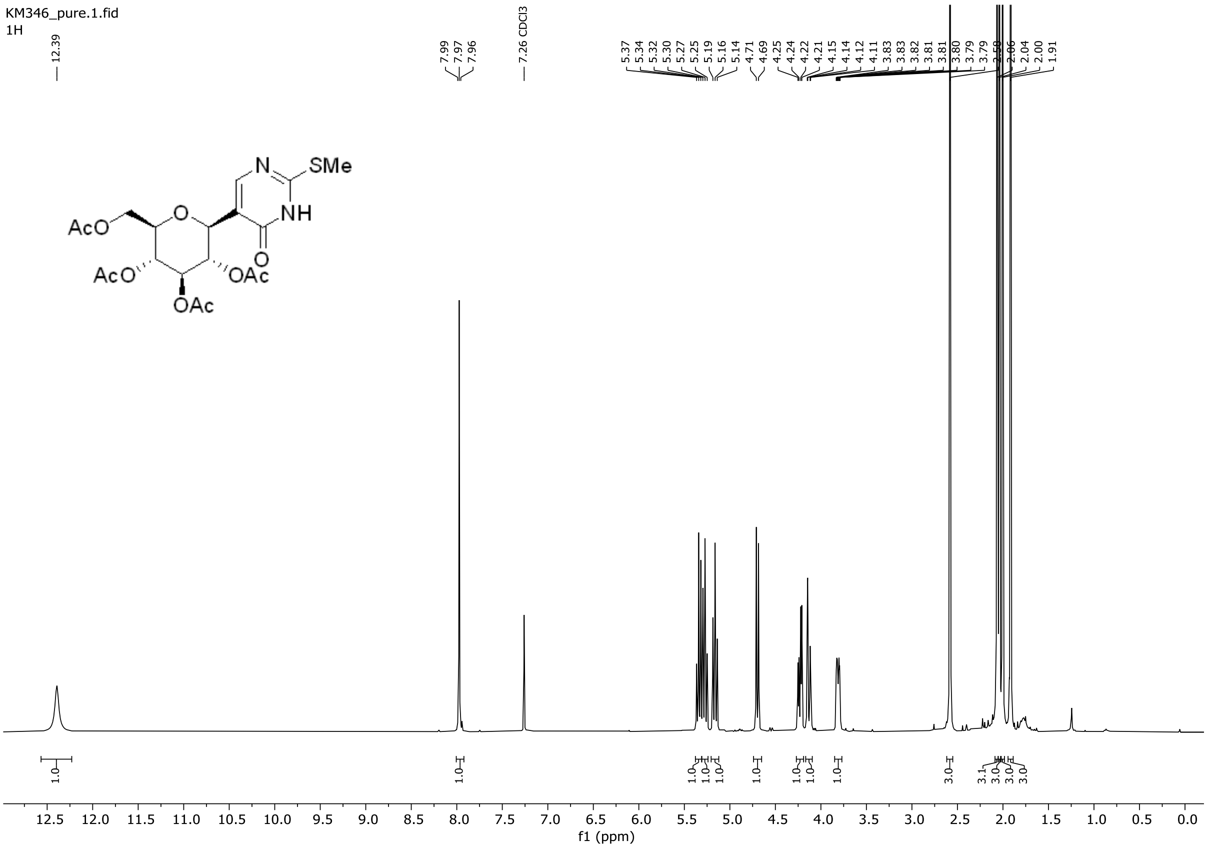

Figure S20. ^{13}C NMR spectrum of compound **16** (100 MHz, $CDCl_3$).

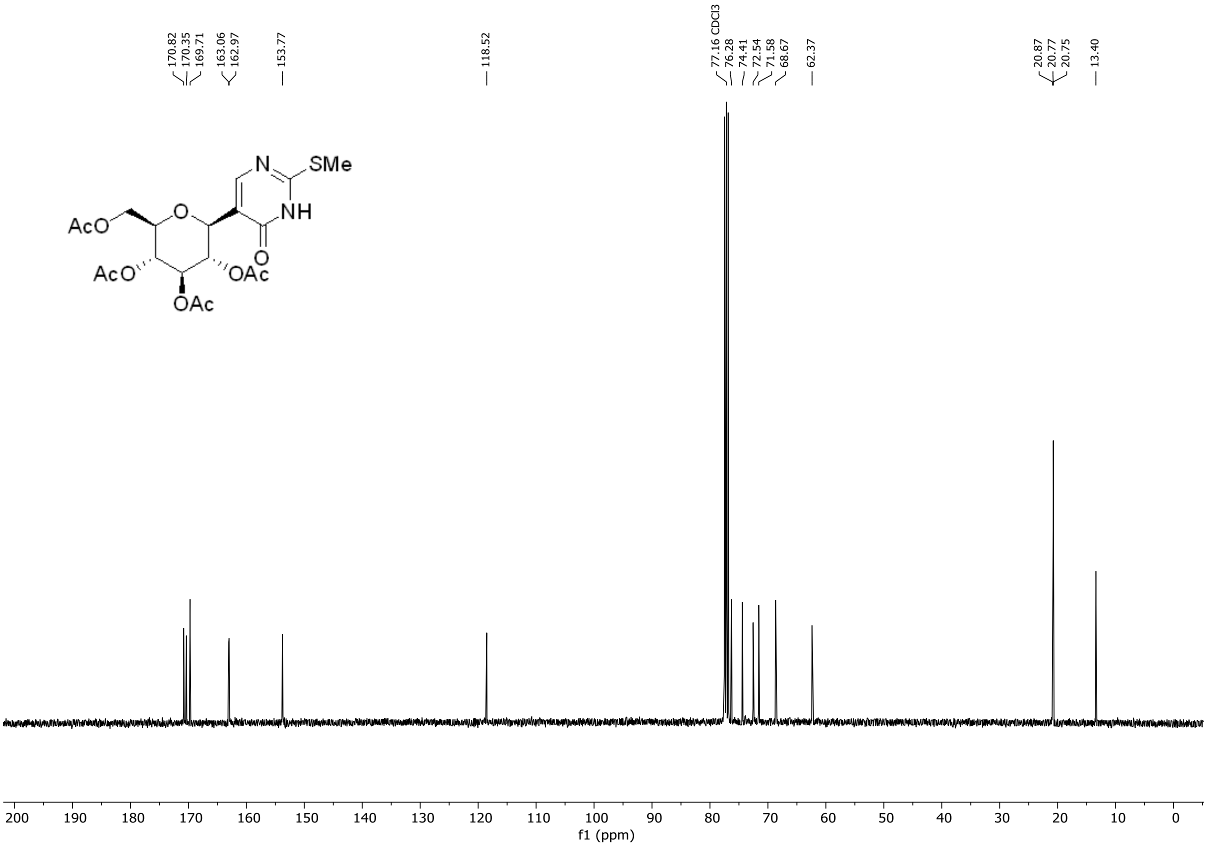

Figure S21. ^{1}H NMR spectrum of compound **6** (400 MHz, DMSO-d$_6$, D$_2$O).

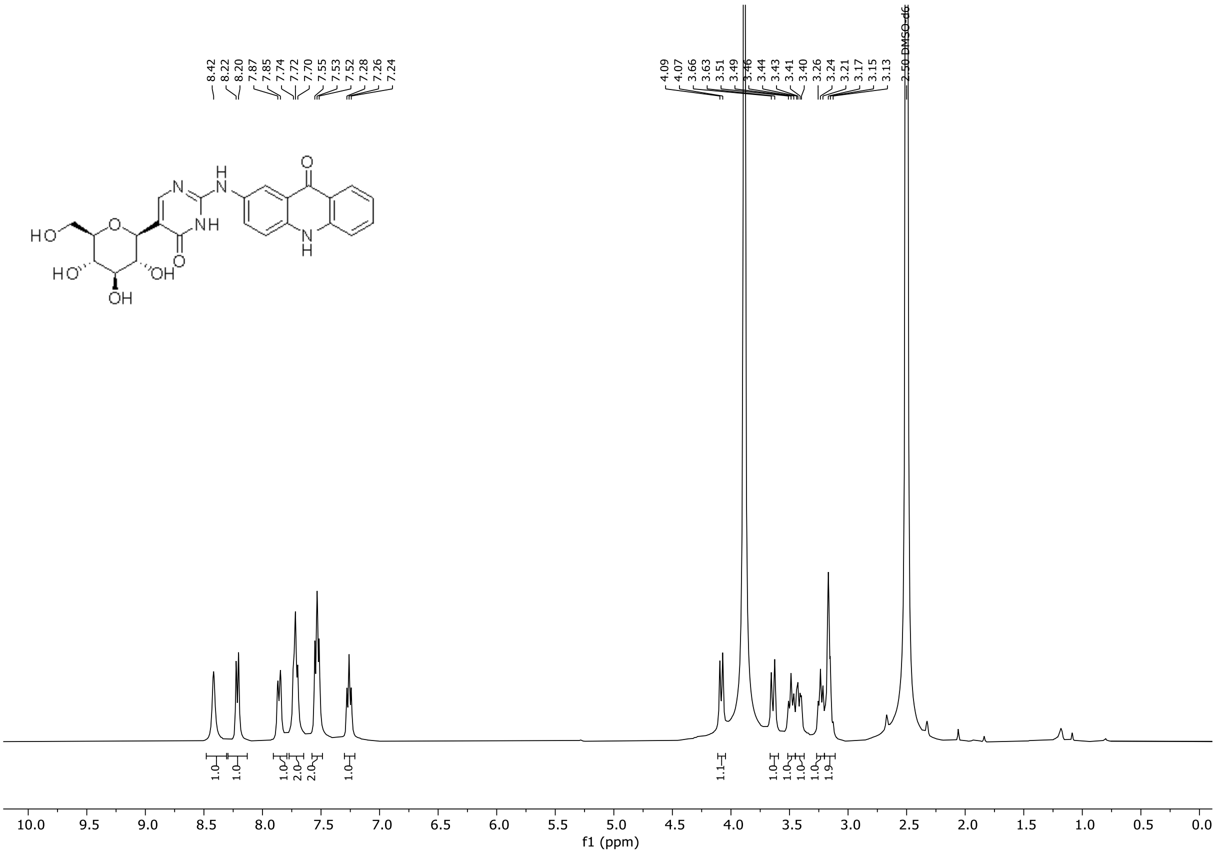

Figure S22. ^{13}C NMR spectrum of compound **6** (100 MHz, DMSO-d$_6$).

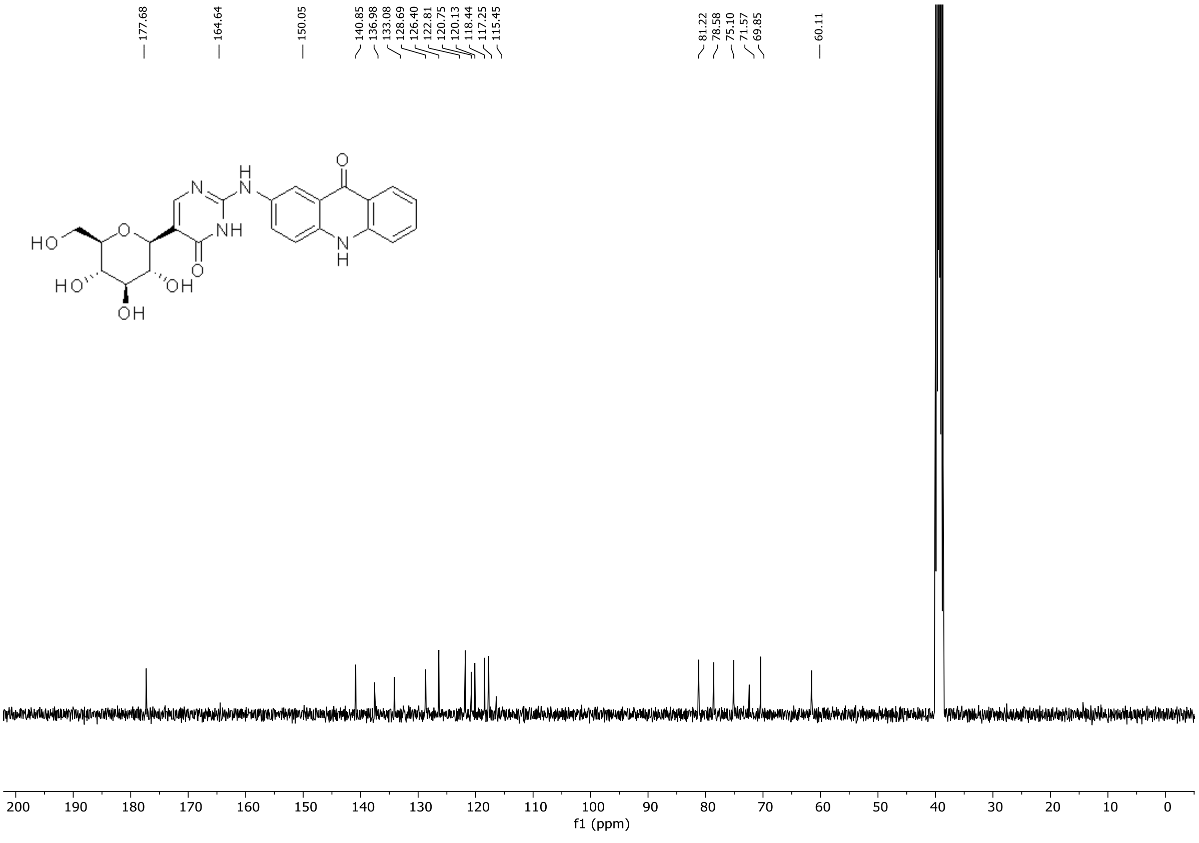

Table S1. Energies E (a.u), free energies ΔG (a.u.) free energy difference ΔΔG (kcal/mol), dihedral angles ψ_1 and ψ_2 (degrees) and % of population of the Ψ-GLAC tautomers and Neutral GLAC.

**Tautomer A**[1]	ψ_1	ψ_2	E	ΔE	ΔG	ΔΔG	%pop
s-cis/s-trans	0.9	141.3	-1635.14849	2.3	-1634.76621	2.6	21.4
s-cis/s-cis	-0.9	0.5	-1635.14839	2.4	-1634.76546	3.1	9.4
s-trans/s-trans	-176.9	-119.6	-1635.14867	2.2	-1634.76664	2.4	28.8
s-trans/s-cis	-178.4	58.8	-1635.14899	2.0	-1634.76692	2.2	40.4
**Tautomer B**[1]	ψ_1	ψ_2	E	ΔE	ΔG	ΔΔG	%pop
s-cis/s-trans	-2.9	119.6	-1635.15201	0.1	-1634.76978	0.4	24.3
s-cis/s-cis	2.4	57.5	-1635.15221	0.0	-1634.77040	0.0	45.9
s-trans/s-trans	179.3	-138.0	-1635.15181	0.2	-1634.76945	0.6	17.4
s-trans/s-cis	179.2	24.6	-1635.15170	0.3	-1634.76913	0.8	12.4
TS (s-cis/s-trans_ s-cis/s-cis)	-4.2	93.6	-1635.15171	0.3	-1634.76738	1.9	
**Tautomer C**[1]	ψ_1	ψ_2	E	ΔE	ΔG	ΔΔG	
s-cis/s-trans	-4.0	119.0	-1635.14712	3.2	-1634.76477	3.5	
s-cis/s-cis	4.2	58.7	-1635.14759	2.9	-1634.76503	3.4	
**Glac Neutral**[2]	ψ_1	ψ_2	E	ΔE	ΔG	ΔΔG	%pop
s-cis/s-trans	2.0	148.0	-1635.14841	0.3	-1634.76618	0.0	50.0
s-cis/s-cis	0.2	2.7	-1635.14886	0.0	-1634.76555	0.4	26.5
s-trans/s-trans	-175.5	-124.0	-1635.14746	0.9	-1634.76497	0.8	13.5
s-trans/s-cis	-175.4	49.7	-1635.14748	0.9	-1634.76460	1.0	10.0
**s-cis/s-trans- conf. locked**[3]	ψ_1	ψ_2	E	ΔE			
Tautomer A	-7.7	168.1	-1635.14789	2.7			
Tautomer B	-7.7	168.1	-1635.14671	3.4			
GLAC	-7.7	168.1	-1635.14815	0.4			

[1]ΔE values refer to s-cis/s-cis minimum of **tautomer B**

[2] ΔE values refer to s-cis/s-cis minimum of neutral GLAC

[3] Minimized with dihedrals ψ_1 = -7.7° and ψ_2 = 168.1°, locked at the conformation of GLAC, found within the catalytic site of GPMM (Maffeis, V.; Mavreas, K.; Monti, F.; Mamais, M.; Gustavsson, T.; Chrysina, E.D.; Markovitsi, D.; Gimisis, T.; Venturini, A. Multiscale time-resolved fluorescence study of a glycogen phosphorylase inhibitor combined with quantum chemistry calculations. *Phys. Chem. Chem. Phys.* **2019**, *21*, 7685–7696).

Table S2. Model Cartesian Coordinates

Tautomer A

s-cis/s-trans

E = -1635.14848848
DG = -1634.766213

0 1

O	-0.10057900	0.32682900	-0.00359200
C	1.29791000	0.45225900	0.19713400
C	1.70454600	1.90599400	-0.10598700
C	0.88963300	2.83741400	0.77999900
C	-0.59595400	2.60408300	0.60199100
C	-0.89023200	1.13448900	0.86502100
C	3.37648700	-0.90870300	-0.43249700
N	3.94602800	-1.91209100	-1.17330800
C	3.26450300	-2.47936200	-2.13133400
C	1.33212200	-1.18869200	-1.68109400
N	3.80735600	-3.45525400	-2.90436300
O	4.03253400	-0.32479000	0.44047400
O	3.07101300	2.18376500	0.11432700
O	1.14940200	4.19389500	0.47454500
O	-1.35005800	3.37618000	1.51435200
C	-2.32998600	0.75480000	0.59066800
O	-2.54749300	-0.61737700	0.85196400
H	3.52273600	1.36140100	0.38211400
H	2.10043400	4.32201100	0.56503600
H	-1.87004800	-1.09577600	0.36175500
H	-1.01516400	4.27802400	1.45594600
H	1.48585700	-2.58613600	-3.18829200
H	0.29734700	-1.00296000	-1.93083900
H	-2.56760700	1.00415000	-0.45156900
H	-2.98574200	1.32793200	1.24561700
H	1.55122400	0.22681800	1.24436400
H	1.45649700	2.10733400	-1.15687900
H	1.15064100	2.62970100	1.82854200
H	-0.87032700	2.84628100	-0.43482200
H	-0.64218500	0.89482300	1.90875300
H	3.18172500	-3.97123500	-3.50686100
C	5.11427200	-3.98637200	-2.78264400
C	5.30018900	-5.33914600	-2.96910200
C	6.23265700	-3.15972900	-2.55121700
C	6.58380500	-5.90191000	-2.93219800
H	4.46081000	-6.00013800	-3.15307500
C	7.49585900	-3.69823900	-2.49080100
H	6.09141800	-2.09799400	-2.41232900
C	7.69252400	-5.07948700	-2.68229000
C	6.75118700	-7.34923100	-3.14422100
H	8.35021500	-3.05702200	-2.30785400
N	8.95328400	-5.61816400	-2.63239800
C	8.13566400	-7.83961300	-3.07137500
O	5.80064500	-8.08969700	-3.36521300
C	9.19917200	-6.95524900	-2.81828700
C	8.40845700	-9.20509800	-3.25808600
C	10.51684300	-7.44751600	-2.75661100
C	9.69765300	-9.68430900	-3.19724300
H	7.56944800	-9.86261700	-3.45089200
C	10.75357200	-8.79103700	-2.94430000
H	11.33252300	-6.76101300	-2.56139600
H	9.90125000	-10.73739300	-3.34195300
H	11.77073800	-9.16085400	-2.89519200
H	9.73657100	-5.00397100	-2.45817600
C	1.97974100	-0.55579600	-0.68562800
N	1.97808500	-2.13658700	-2.42839300

s-cis/s-cis

E = -1635.14838635
DG = -1634.765459

0 1

O	-0.21192300	0.32098900	-0.12951700
C	1.16545300	0.34353200	0.20832300
C	1.62486100	1.81120300	0.27705900
C	0.75583800	2.54839300	1.28595700
C	-0.71277400	2.42037900	0.93989700
C	-1.05921500	0.94228100	0.83336300
C	3.26395200	-0.92065000	-0.53834400
N	3.88311400	-1.74609000	-1.44328300
C	3.28038200	-2.06543700	-2.55569100
C	1.33640900	-0.83884100	-1.98250000
N	3.85988500	-2.86571000	-3.48590700
O	3.85127900	-0.57915300	0.49616700
O	2.97385300	1.98022000	0.65774800
O	1.06858400	3.92740400	1.32194400
O	-1.52868700	2.99433000	1.94092200
C	-2.47638000	0.69371500	0.36181200
O	-2.74430100	-0.69193300	0.27972400
H	3.38098700	1.10294500	0.78263300
H	2.01063700	3.99344700	1.51509500
H	-2.03600800	-1.07217400	-0.25117900
H	-1.17238500	3.87210500	2.11815600
H	1.58330100	-1.88458300	-3.73728300
H	0.33123400	-0.56291500	-2.26782000
H	-2.61541600	1.18440200	-0.61014300
H	-3.17582200	1.12778700	1.07586200
H	1.32037100	-0.12082400	1.19431000
H	1.47027800	2.25388700	-0.71609600
H	0.92047200	2.09816800	2.27631200
H	-0.89003700	2.90240800	-0.03227900
H	-0.91000600	0.46218300	1.81085400
H	3.31119000	-3.06212000	-4.31112300
C	5.13286400	-3.47346800	-3.47692300
C	6.06600000	-3.36732400	-2.46358900
C	5.44752700	-4.24956200	-4.61548300
C	7.30440000	-4.02201400	-2.57155300
H	5.86944500	-2.78682100	-1.57737300
C	6.65258700	-4.89433400	-4.73214700
H	4.72192000	-4.33882000	-5.41681400
C	7.60815600	-4.78991600	-3.70350600
C	8.28027100	-3.88976500	-1.47521000
H	6.87127900	-5.48345400	-5.61480600
N	8.81806600	-5.43002700	-3.80794900
C	9.55070400	-4.60511800	-1.67053800
O	8.04824000	-3.22899600	-0.47007400
C	9.78236000	-5.35717600	-2.83578200
C	10.55086700	-4.54108900	-0.68569400
C	11.00703500	-6.03322100	-2.99902800
C	11.74738200	-5.20253300	-0.84709200
H	10.34465700	-3.95366700	0.20076800
C	11.96807100	-5.95205100	-2.01621200
H	11.18011500	-6.61125600	-3.89952100
H	12.51372000	-5.14885600	-0.08466900
H	12.90733200	-6.47519900	-2.15027700
H	9.00647500	-5.97807900	-4.63564500
C	1.90403600	-0.46701000	-0.81988100
N	2.02576800	-1.62169900	-2.86744200

s-trans/s-trans

E = -1635.14867132
DG = -1634.766636

0 1

O	0.09594000	0.17695800	-0.00089200
C	1.51427900	0.15160800	0.00899300
C	2.02641100	1.60347100	-0.00375700
C	1.43725700	2.33947600	1.19104500
C	-0.07480700	2.26234300	1.19181600
C	-0.48533000	0.79768700	1.14243200
C	3.34733800	-1.14698200	-1.22625500
N	3.72094900	-1.95103900	-2.26694000

C	2.85718000	-2.23081200	-3.21228800
C	1.13123100	-0.99362600	-2.17232000
N	3.24853000	-3.01841100	-4.24114000
O	4.15954500	-0.84354600	-0.34022400
O	3.43126100	1.72436100	0.06187800
O	1.79604500	3.70774800	1.17878200
O	-0.61782300	2.83482100	2.36436300
C	-1.98044000	0.60091300	1.00722300
O	-2.30574100	-0.77427200	0.96626700
H	3.82587400	0.83137800	0.06340200
H	2.75888800	3.74174900	1.14827100
H	-1.75115800	-1.16179400	0.28041600
H	-0.19583800	3.69454200	2.47258400
H	0.93014400	-2.06322800	-3.93182300
H	0.09249200	-0.69822000	-2.20600500
H	-2.32343300	1.11839200	0.10197000
H	-2.48155800	1.03744300	1.87085700
H	1.87680700	-0.34118000	0.92398700
H	1.66309400	2.07513600	-0.92675500
H	1.81041700	1.85965300	2.10831100
H	-0.45550300	2.77309100	0.29568400
H	-0.13103800	0.28985000	2.05065800
H	4.17309900	-3.41335500	-4.13874100
C	2.46007300	-3.36462600	-5.37255700
C	2.16297200	-4.68385800	-5.63847800
C	2.00698000	-2.35842800	-6.24900800
C	1.41935700	-5.03312900	-6.77302300
H	2.50258900	-5.47431100	-4.97996200
C	1.25075800	-2.67703900	-7.35178700
H	2.26628300	-1.32435200	-6.05159300
C	0.94941300	-4.02453000	-7.63141100
C	1.12465900	-6.45222200	-7.04422000
H	0.90014800	-1.89981000	-8.02002800
N	0.20908100	-4.35198600	-8.73501100
C	0.32572300	-6.70911000	-8.25214100
O	1.51992400	-7.34891100	-6.31154500
C	-0.10917500	-5.64794900	-9.06457000
C	-0.00986800	-8.02638800	-8.60511600
C	-0.87177100	-5.91679800	-10.21596500
C	-0.75606400	-8.28786500	-9.73252900
H	0.33927000	-8.82362700	-7.96026800
C	-1.18583000	-7.21907200	-10.53754500
H	-1.20546800	-5.09509100	-10.83899100
H	-1.01177600	-9.30498900	-9.99972400
H	-1.77370200	-7.41725600	-11.42570100
H	-0.11357000	-3.60767600	-9.33791100
C	1.96808700	-0.65420700	-1.17622500
N	1.57689400	-1.77186400	-3.21047100

s-trans/s-cis

E = -1635.14898674
DG = -1634.766923

0 1

O	0.08114100	0.15886600	-0.12488500
C	1.49494100	0.11878900	-0.01527000
C	2.01516200	1.56373000	0.09264700
C	1.34688000	2.23556100	1.28367300
C	-0.16189900	2.17380100	1.17233900
C	-0.57702500	0.71897200	1.00800100
C	3.40243700	-1.12981800	-1.18745700
N	3.84098100	-1.88431600	-2.24013600
C	3.04622900	-2.09896700	-3.26010300
C	1.26425200	-0.89278100	-2.28242000
N	3.50777300	-2.84342600	-4.29352600
O	4.15257700	-0.88388300	-0.23170600
O	3.41262900	1.66628500	0.26362200
O	1.71417200	3.59840000	1.37673900
O	-0.78445000	2.68182000	2.33504300
C	-2.05977100	0.54665300	0.75469200
O	-2.39090400	-0.82029400	0.61164400
H	3.80051900	0.77062900	0.24371400
H	2.67710700	3.62410700	1.41254000
H	-1.79166600	-1.17431300	-0.05447800
H	-0.37148000	3.53272000	2.52022900
H	1.18425000	-1.85460700	-4.11323500
H	0.23417700	-0.58015400	-2.37510800
H	-2.33296000	1.11828300	-0.14165800
H	-2.61901400	0.93913400	1.60366000
H	1.78788000	-0.42927400	0.89323600
H	1.72117600	2.09125600	-0.82477100
H	1.65046800	1.69985900	2.19566800
H	-0.47420800	2.73995100	0.28308600
H	-0.29287300	0.15572100	1.90821700
H	4.41258500	-3.26244700	-4.12707700
C	2.81137700	-3.15479200	-5.49114900
C	2.35122500	-2.16068100	-6.32905200
C	2.61393400	-4.50504400	-5.84036500
C	1.66219400	-2.48191600	-7.50683900
H	2.51552200	-1.11261900	-6.10401100
C	1.96920500	-4.84101600	-7.00573900
H	2.97946600	-5.28027800	-5.17768500
C	1.47468200	-3.82961700	-7.85401100
C	1.16675800	-1.40135600	-8.37835400
H	1.82494200	-5.88118200	-7.27238500
N	0.81659200	-4.15574200	-9.00899000
C	0.47024600	-1.84503800	-9.59501800
O	1.32580700	-0.21987000	-8.10160800
C	0.31498700	-3.21363400	-9.87459500
C	-0.05026100	-0.89782600	-10.49205500
C	-0.35609300	-3.61635200	-11.04381500
C	-0.70680700	-1.29361600	-11.63582400
H	0.08373600	0.14956000	-10.25060100
C	-0.85593200	-2.66494800	-11.90555700
H	-0.47325200	-4.67318700	-11.25363500
H	-1.10603800	-0.55941100	-12.32348700
H	-1.37152400	-2.98344600	-12.80355400
H	0.69564000	-5.13276600	-9.23766100
C	2.02789300	-0.62299700	-1.20922500
N	1.77709100	-1.61713200	-3.32846100

s-cis/s-trans with conformationally locked dihedrals at ψ_1, ψ_2

E = -1635.14789447

0 1

O	-1.50444200	-4.00150200	-5.50763700
C	-0.76414500	-5.13741700	-5.09090500
C	-1.65768500	-5.98853500	-4.17082800
C	-2.92591100	-6.35581200	-4.92779000
C	-3.63738700	-5.11981400	-5.43637400
C	-2.66226800	-4.31034800	-6.27916300
C	1.60454800	-5.57454000	-4.22105600
N	2.77621700	-5.09051600	-3.69711600
C	2.87100400	-3.83294400	-3.35993200
C	0.65505400	-3.36507200	-4.05585100
N	3.99980900	-3.31793900	-2.81036900
O	1.50029900	-6.77280600	-4.51383900
O	-1.05683900	-7.18435700	-3.72107000
O	-3.83111900	-7.06134300	-4.10110600
O	-4.74366400	-5.46078700	-6.24706900
C	-3.22683600	-2.98065400	-6.73253900
O	-2.28376800	-2.27209000	-7.51166300
H	-0.14791900	-7.23399900	-4.07136200
H	-3.35537800	-7.82673100	-3.75898200
H	-1.47258600	-2.24337400	-6.99278500
H	-5.25153300	-6.11862700	-5.75901200
H	1.94868200	-1.97863400	-3.25401800
H	-0.10522800	-2.60794400	-4.18470100
H	-3.52543100	-2.40443700	-5.84729500
H	-4.10774600	-3.15444600	-7.35009000
H	-0.47959100	-5.74411800	-5.96420200
H	-1.92655000	-5.36988700	-3.30419000
H	-2.64321800	-6.97480500	-5.79254500
H	-3.95620700	-4.51581200	-4.57477600
H	-2.36586200	-4.89967300	-7.15842700
H	3.95134000	-2.35856400	-2.49711700
C	5.26536300	-3.92069600	-2.63756300

C	6.16876900	-3.26370900	-1.82297900
C	5.66464900	-5.11263900	-3.27819000
C	7.46113400	-3.76072900	-1.62264700
H	5.90115400	-2.34220400	-1.31734200
C	6.93101800	-5.61614400	-3.08183100
H	4.96943000	-5.63709300	-3.91374300
C	7.85057300	-4.95246600	-2.25211900
C	8.39572600	-3.03026300	-0.74921800
H	7.22609100	-6.53338900	-3.57800800
N	9.11359600	-5.45554900	-2.05936900
C	9.72574300	-3.63903800	-0.60317800
O	8.08184000	-1.98845300	-0.18656600
C	10.04613800	-4.83779400	-1.26590800
C	10.69424100	-3.02158400	0.20610800
C	11.32862700	-5.39857600	-1.11033400
C	11.94667700	-3.57234000	0.35721300
H	10.41768500	-2.09990000	0.70363200
C	12.25696900	-4.77037000	-0.31053900
H	11.57220100	-6.32132000	-1.62407400
H	12.68831400	-3.09203600	0.98222200
H	13.24077800	-5.20983900	-0.19725600
H	9.36579500	-6.31740200	-2.52271800
C	0.49160000	-4.64913200	-4.42444500
N	1.83833400	-2.95092800	-3.50702400

Tautomer B

s-cis/s-trans

E = -1635.152009
DG = -1634.769776

0 1

O	-0.07627500	0.27957600	-0.08067000
C	1.32224900	0.27805600	0.15079700
C	1.83759900	1.71988400	0.01521100
C	1.10131700	2.59558800	1.01833700
C	-0.39709400	2.49873100	0.81268400
C	-0.81318800	1.03516700	0.87726900
C	3.28570900	-1.15039400	-0.49918700
N	3.78283600	-2.07517200	-1.41270400
C	3.10380000	-2.47405600	-2.52387700
C	1.35794200	-1.15314900	-1.93655300
N	3.69644500	-3.36252800	-3.35413100
O	3.96818100	-0.82471800	0.47175400
O	3.23150300	1.85368300	0.21288500
O	1.46359700	3.95594200	0.88247800
O	-1.10093900	3.19995500	1.81780300
C	-2.27404800	0.81784700	0.54343800
O	-2.60846400	-0.55369700	0.61828900
H	3.58073700	1.03448500	0.59755300
H	2.42268100	3.99824600	0.96921500
H	-1.96226700	-1.01798100	0.07501600
H	-0.70699400	4.07792300	1.87019900
H	0.35190800	-0.82252000	-2.17016900
H	-2.47020900	1.22126900	-0.45839800
H	-2.89285200	1.35287200	1.26341800
H	1.52860000	-0.06990000	1.17543000
H	1.60420900	2.06625900	-0.99896400
H	1.33825100	2.24064700	2.03279000
H	-0.63937100	2.89720000	-0.18311300
H	-0.60696500	0.64362200	1.88360600
H	3.09950900	-3.70496800	-4.09446500
C	5.00784300	-3.89284900	-3.21426100
C	5.19772800	-5.24988600	-3.06759600
C	6.12446000	-3.03452300	-3.26243400
C	6.48920200	-5.78313200	-2.97018200
H	4.35433700	-5.92902400	-3.03048200
C	7.39921500	-3.53417600	-3.13914700
H	5.97265100	-1.97077900	-3.40720700
C	7.59917700	-4.92148400	-2.99492700
C	6.66729600	-7.23962400	-2.82559300
H	8.25416400	-2.86956500	-3.17325000
N	8.86521300	-5.43060500	-2.88651400
C	8.06157600	-7.69562900	-2.72187500
O	5.71710500	-8.01000100	-2.79517600
C	9.12239200	-6.77450500	-2.75716200
C	8.34511900	-9.06462600	-2.58809600
C	10.44908600	-7.23270300	-2.66054000
C	9.64407800	-9.51136600	-2.49209600
H	7.50757200	-9.75110500	-2.56409200
C	10.69723300	-8.58149000	-2.52990500
H	11.26341900	-6.51816400	-2.69140300
H	9.85690200	-10.56749900	-2.38916200
H	11.72177700	-8.92582800	-2.45614500
H	9.64710400	-4.79048000	-2.90317300
C	1.96883100	-0.67495200	-0.81282700
N	1.90005300	-2.03536100	-2.81148600
H	4.69527000	-2.46553400	-1.19966700

s-cis/s-cis

E = -1635.15220782
DG = -1634.770395

0 1

O	-0.04863600	0.15851300	0.17228900
C	1.33453500	0.45437600	0.07626700
C	1.48809700	1.92770200	-0.33551400
C	0.79272500	2.79767200	0.70122500
C	-0.65962300	2.39215000	0.85139800
C	-0.72629800	0.90646200	1.17865500
C	3.38634500	-0.66837600	-0.84886800
N	3.87370900	-1.61816400	-1.74130200
C	3.08410000	-2.31008700	-2.60799800
C	1.25466100	-1.24186400	-1.80046800
N	3.66529600	-3.19621800	-3.45065500
O	4.16823700	-0.06351200	-0.11562000
O	2.83039600	2.35151900	-0.47115600
O	0.81922600	4.16300600	0.33347100
O	-1.28657600	3.10197400	1.90041100
C	-2.14130600	0.36870500	1.21386100
O	-2.14779200	-1.01073900	1.52295700
H	3.42017600	1.67323000	-0.10564000
H	1.74428900	4.39541600	0.19412700
H	-1.53701600	-1.42751300	0.90532300
H	-1.08821800	4.03463000	1.76046800
H	0.17680900	-1.13078200	-1.84351000
H	-2.61551600	0.56130100	0.24281300
H	-2.70594900	0.88772100	1.98801400
H	1.81338700	0.31631700	1.05868400
H	0.98944000	2.05910100	-1.30367000
H	1.29637400	2.65698700	1.66963400
H	-1.17372200	2.57297400	-0.10360600
H	-0.24362900	0.72720600	2.14998900
H	3.03436600	-3.58078700	-4.14096400
C	5.04194000	-3.53673800	-3.51012900
C	5.70550700	-4.03231000	-2.40735000
C	5.73277100	-3.39003500	-4.72902900
C	7.06706700	-4.35769600	-2.48009500
H	5.19432200	-4.18800600	-1.46373500
C	7.05871000	-3.73379500	-4.82950100
H	5.20447300	-3.00218800	-5.59167700
C	7.74949400	-4.21490400	-3.69899100
C	7.75811000	-4.87008400	-1.28334200
H	7.58399100	-3.62466200	-5.77069100
N	9.07562100	-4.54260200	-3.78620000
C	9.17995100	-5.19565500	-1.47000000
O	7.18297500	-5.01155900	-0.21238200
C	9.79852400	-5.02080800	-2.71990100
C	9.93602200	-5.68547900	-0.39233600
C	11.16055000	-5.33755200	-2.87598600
C	11.26892200	-5.99451900	-0.54671800
H	9.43293600	-5.80956600	0.55890700
C	11.87709100	-5.81622200	-1.80126300
H	11.63223100	-5.20161400	-3.84224900
H	11.84681100	-6.37146700	0.28710100
H	12.92538100	-6.05748200	-1.92992900
H	9.54447700	-4.42820800	-4.67427800
C	1.96222100	-0.50171900	-0.89711100

N	1.78298700	-2.14258800	-2.66490900
H	4.88099300	-1.74266300	-1.75293900

s-trans/s-trans

E = -1635.15181105
DG = -1634.769446

0 1

O	0.06929900	0.38214100	0.11831900
C	1.47741800	0.22346100	0.09154900
C	2.12118600	1.61853800	0.03637300
C	1.66741300	2.41047600	1.25393600
C	0.15437800	2.47793800	1.31297100
C	-0.40696400	1.06274100	1.27685200
C	3.13367500	-1.27402100	-1.06680500
N	3.36467200	-2.09848100	-2.15988400
C	2.46427700	-2.28782700	-3.17035600
C	1.00868600	-0.89712100	-2.12686900
N	2.86001900	-3.11482400	-4.17157100
O	3.99979600	-1.14215000	-0.20334700
O	3.53429400	1.60013100	-0.01006300
O	2.15312500	3.73804600	1.21827700
O	-0.28748400	3.09456800	2.50529800
C	-1.91813900	1.02492500	1.18908400
O	-2.38761600	-0.30826000	1.16351700
H	3.85075000	0.71143500	0.21548900
H	3.11127600	3.68116700	1.12918400
H	-1.90003100	-0.75212600	0.46108100
H	0.20444600	3.91891900	2.58984000
H	0.02391600	-0.44354000	-2.14094200
H	-2.23276400	1.57494000	0.29278700
H	-2.34350600	1.51237800	2.06599700
H	1.81249000	-0.27870900	1.01295800
H	1.75969000	2.11951800	-0.86979800
H	2.02985700	1.89923000	2.15866200
H	-0.20496800	3.02961800	0.43232100
H	-0.08040900	0.52011900	2.17544100
H	3.68864800	-3.67148500	-4.01554000
C	2.08465600	-3.46225900	-5.30525800
C	2.08354100	-4.76901700	-5.74166800
C	1.37341500	-2.48731000	-6.03356500
C	1.38199700	-5.14039200	-6.89783800
H	2.62536200	-5.53951200	-5.20510700
C	0.66299500	-2.83725000	-7.15651200
H	1.38534800	-1.45953700	-5.70019400
C	0.65576100	-4.17169100	-7.60686800
C	1.40337700	-6.54290200	-7.34525300
H	0.11534400	-2.08389500	-7.71033600
N	-0.04766100	-4.52255500	-8.73078500
C	0.62151100	-6.82844100	-8.55771300
O	2.02948700	-7.40869300	-6.74610900
C	-0.08299300	-5.80542900	-9.21664100
C	0.57323800	-8.13525800	-9.07101800
C	-0.82414100	-6.10289500	-10.37605500
C	-0.15227200	-8.42418800	-10.20488300
H	1.12615300	-8.90235300	-8.54260800
C	-0.85327100	-7.39362500	-10.85548200
H	-1.36535300	-5.31049400	-10.87998900
H	-0.18565700	-9.43307400	-10.59528500
H	-1.42666700	-7.61343600	-11.74807900
H	-0.56191600	-3.80543500	-9.22321700
C	1.84194400	-0.64337200	-1.07882200
N	1.29326200	-1.70571800	-3.18150600
H	4.27228300	-2.55068300	-2.18308400

s-trans/s-cis

E = -1635.15169512
DG = -1634.769130

0 1

O	0.03170200	0.38997600	-0.13955300
C	1.40887800	0.11512800	0.04991300
C	2.14611400	1.44967300	0.24760100
C	1.54444600	2.16150100	1.45077700
C	0.05174700	2.35093400	1.26778300
C	-0.59014200	0.99820600	0.98966300
C	3.13459900	-1.40527900	-0.97165300
N	3.48997800	-2.14658500	-2.08958200
C	2.76486400	-2.16604800	-3.25074800
C	1.25103700	-0.75704300	-2.31752600
N	3.27089500	-2.94215700	-4.24195500
O	3.84679400	-1.42533400	0.03088300
O	3.54163500	1.31643300	0.43184800
O	2.11738100	3.44109200	1.63492600
O	-0.54527800	2.88890300	2.43032700
C	-2.06220000	1.09847600	0.64933800
O	-2.60990100	-0.18094700	0.40154000
H	3.75370700	0.38755900	0.61334400
H	3.07057900	3.31258700	1.69895400
H	-2.03936600	-0.59541700	-0.25490600
H	-0.02131700	3.65814800	2.68030200
H	0.31587000	-0.22370400	-2.44579500
H	-2.17823400	1.75659700	-0.22145700
H	-2.59883400	1.53467600	1.49148100
H	1.54499600	-0.49556700	0.95646600
H	1.98028300	2.06072500	-0.64784700
H	1.71062500	1.54027800	2.34398200
H	-0.11283200	3.01095400	0.40389600
H	-0.46154500	0.34845200	1.86703700
H	4.08127900	-3.49894500	-4.01089400
C	2.74051100	-3.16997700	-5.53051600
C	1.89321600	-2.30280200	-6.19076300
C	3.15428900	-4.35274500	-6.17982000
C	1.43782200	-2.60548500	-7.48353100
H	1.55393200	-1.38796200	-5.73161800
C	2.72827400	-4.65503600	-7.44928400
H	3.82022000	-5.03722600	-5.66577900
C	1.85346000	-3.78228600	-8.12320300
C	0.52706300	-1.66625800	-8.16239900
H	3.05709000	-5.56740900	-7.93227300
N	1.41278800	-4.07810600	-9.38876900
C	0.10550800	-2.06196500	-9.51495500
O	0.14839700	-0.62775000	-7.63455600
C	0.56264300	-3.26117600	-10.08919500
C	-0.76571100	-1.23911300	-10.24845300
C	0.14421200	-3.61929900	-11.38508500
C	-1.17371800	-1.59095900	-11.51544200
H	-1.10228000	-0.32202400	-9.78035000
C	-0.70966200	-2.79226200	-12.08002600
H	0.50045300	-4.54502000	-11.82193600
H	-1.84484000	-0.95251300	-12.07526400
H	-1.02625900	-3.07585400	-13.07662200
H	1.72668700	-4.93454400	-9.82377400
C	1.90904400	-0.66923200	-1.12842100
N	1.65838400	-1.48482600	-3.39175000
H	4.35068600	-2.67667500	-2.00445500

TS s-cis/s-trans_s-cis/s-cis

E = -1635.15171274
DG = -1634.767378

0 1

O	-0.87541600	-0.45884600	-2.59317400
C	-0.27099500	-1.49902200	-1.84280800
C	-0.68327700	-1.33661400	-0.37063600
C	-2.20156400	-1.38354200	-0.28321300
C	-2.82130600	-0.32428100	-1.17209600
C	-2.29996400	-0.50372000	-2.59148700
C	1.98146200	-2.61219100	-1.73823500
N	3.33756500	-2.49007800	-2.02169400
C	3.90152500	-1.35817500	-2.52759700
C	1.86862900	-0.35878300	-2.57002200
N	5.23389300	-1.36200500	-2.75036000
O	1.54910200	-3.66273000	-1.26336700

O	-0.13866000	-2.31574800	0.49225800
O	-2.65190200	-1.15020800	1.03703500
O	-4.22972400	-0.43812500	-1.20333900
C	-2.75102800	0.59271100	-3.53335000
O	-2.24278200	0.37844000	-4.83499300
H	0.25887400	-3.02516700	-0.03724200
H	-2.21320600	-1.79909400	1.59889400
H	-1.29288100	0.25203400	-4.73526600
H	-4.52315700	-0.49309900	-0.28700600
H	1.29734300	0.52560400	-2.82994500
H	-2.42150700	1.55965200	-3.13165800
H	-3.83899800	0.59175000	-3.59472000
H	-0.63627700	-2.47508900	-2.19993800
H	-0.33665900	-0.35275000	-0.03131700
H	-2.54083100	-2.37162900	-0.62938100
H	-2.51834200	0.66522000	-0.80042900
H	-2.63232600	-1.47730900	-2.97909900
H	5.60935700	-0.49268300	-3.10403200
C	6.11125800	-2.43675700	-2.41433500
C	6.41821000	-3.40510800	-3.34540400
C	6.66466400	-2.50935600	-1.12138300
C	7.28128500	-4.45957500	-3.01968400
H	6.00059500	-3.36672800	-4.34447900
C	7.51211000	-3.53551000	-0.77856200
H	6.41509600	-1.74231800	-0.39791200
C	7.83334200	-4.52676600	-1.72821700
C	7.60033900	-5.48163200	-4.03311500
H	7.93755300	-3.58885100	0.21634700
N	8.67707300	-5.55111700	-1.39803000
C	8.51542500	-6.54394400	-3.58803400
O	7.13387700	-5.44348400	-5.16338100
C	9.02771600	-6.54697300	-2.27980400
C	8.88622000	-7.56895400	-4.47306400
C	9.90112200	-7.57157800	-1.87353100
C	9.74201200	-8.57162200	-4.07415400
H	8.47591000	-7.54069100	-5.47512500
C	10.24800600	-8.56492400	-2.76328300
H	10.29244400	-7.56879300	-0.86289300
H	10.02480100	-9.35925200	-4.76043900
H	10.92147000	-9.35053300	-2.44248400
H	9.06356400	-5.57929900	-0.46443700
C	1.21488800	-1.44146500	-2.05413000
N	3.20076400	-0.28290000	-2.80753800
H	3.91400400	-3.30189400	-1.82364700

s-cis/s-trans with conformationally locked dihedrals at ψ_1, ψ_2

E = -1635.14671118

0 1

O	3.55687100	1.67436900	-7.90621700
C	3.98763000	0.34895600	-8.16521900
C	2.92787500	-0.34253800	-9.03860800
C	2.76098600	0.45624900	-10.32290700
C	2.40044200	1.89548900	-10.01351100
C	3.45036800	2.47953800	-9.07774800
C	5.03906200	-1.51936700	-6.85187400
N	5.26086800	-2.05522600	-5.58251300
C	4.70236000	-1.55189600	-4.44818800
C	3.69996300	0.08247800	-5.66491000
N	4.95295800	-2.12598100	-3.24559900
O	5.53438200	-2.06888000	-7.83419300
O	3.23232000	-1.68519600	-9.36109800
O	1.73246600	-0.07668800	-11.13398000
O	2.37211000	2.68283700	-11.18678100
C	3.10650500	3.87299200	-8.59520800
O	4.11044600	4.36877000	-7.73224600
H	4.15560800	-1.87052700	-9.12879300
H	1.94831800	-1.00424800	-11.28303700
H	4.23962400	3.69555500	-7.05529000
H	1.82886700	2.20819200	-11.82581300
H	3.06832900	0.96383500	-5.65059700
H	2.13050000	3.84157600	-8.09394100
H	3.03890000	4.54484600	-9.45050400
H	4.93868500	0.36866500	-8.72089500
H	1.98003400	-0.33240300	-8.48669000
H	3.71696000	0.44507500	-10.86809000
H	1.42280500	1.91020900	-9.51050400
H	4.42201100	2.50097700	-9.59141700
H	4.40450000	-1.69543500	-2.51211700
C	5.83724800	-3.14266400	-2.83153000
C	5.65209600	-3.65799900	-1.56040500
C	6.98459600	-3.53897300	-3.55147800
C	6.51436400	-4.62121800	-1.03089700
H	4.81660300	-3.33875700	-0.94753000
C	7.83682000	-4.49767000	-3.05027900
H	7.26577800	-3.06374300	-4.48028600
C	7.60911800	-5.06793300	-1.78709300
C	6.26318700	-5.15784400	0.32037100
H	8.70920400	-4.78904500	-3.62276700
N	8.45389400	-6.02378000	-1.28268400
C	7.21997600	-6.17410400	0.78023000
O	5.32118200	-4.77886800	1.00365500
C	8.29042100	-6.57746100	-0.03787600
C	7.07095500	-6.75368900	2.05148500
C	9.19486700	-7.55131300	0.42654500
C	7.95612100	-7.70623900	2.50300500
H	6.23702100	-6.42334500	2.65885800
C	9.02335100	-8.10154200	1.67718300
H	10.01877800	-7.85731400	-0.20761700
H	7.83599900	-8.14931500	3.48308100
H	9.72427700	-8.85043400	2.02595100
H	9.23597400	-6.32700500	-1.84622500
C	4.21915500	-0.34412400	-6.85374600
N	3.92115200	-0.49557900	-4.46025300
H	5.73238200	-2.95125100	-5.57630500

Tautomer C

s-cis/s-trans

E = -1635.14712396
DG = -1634.764766

0 1

O	-0.07171200	0.27524800	-0.09206500
C	1.32407000	0.25927300	0.14954200
C	1.85174300	1.69794200	0.03213500
C	1.12231200	2.56521000	1.04787800
C	-0.37663100	2.48430200	0.83486800
C	-0.80875700	1.02427700	0.87266700
C	3.28642300	-1.19903600	-0.51260700
N	3.79945500	-2.10436600	-1.42850200
C	3.19190300	-2.54380500	-2.59167600
C	1.35028500	-1.12314400	-1.94172600
N	3.66569700	-3.37570100	-3.43543000
O	3.93809300	-0.89547400	0.47824800
O	3.24826800	1.81543800	0.21781100
O	1.49564200	3.92376800	0.93141500
O	-1.07854900	3.17426100	1.84857700
C	-2.26921700	0.82853900	0.52465800
O	-2.62240700	-0.53888300	0.58252300
H	3.57714700	1.02491800	0.67017200
H	2.45520100	3.95791400	1.01659600
H	-1.98462000	-1.00645500	0.03240000
H	-0.68002100	4.04917000	1.91551000
H	0.35294700	-0.79983400	-2.20813200
H	-2.45242900	1.24557400	-0.47400400
H	-2.88616400	1.36392700	1.24589500
H	1.52230500	-0.10601100	1.16911600
H	1.62039100	2.05972000	-0.97642100
H	1.35257100	2.19403300	2.05809800
H	-0.60996900	2.90237600	-0.15502800
H	-0.61411000	0.61372900	1.87349200
C	4.97069000	-3.87754900	-3.25941800
C	5.17832600	-5.23315700	-3.08646000
C	6.09973900	-3.02931000	-3.33573900

C 6.47150900 -5.76278400 -2.97088800
H 4.33642500 -5.91299400 -3.04020500
C 7.37799300 -3.52689500 -3.22434900
H 5.95068300 -1.96739500 -3.49962900
C 7.58218900 -4.90633300 -3.03707600
C 6.65348600 -7.21316700 -2.78504600
H 8.23210900 -2.86261400 -3.28863100
N 8.85463500 -5.41325000 -2.92876700
C 8.04872700 -7.66693000 -2.68238200
O 5.70540500 -7.98653300 -2.72211700
C 9.11144100 -6.74912200 -2.75933500
C 8.33236300 -9.03184100 -2.50713200
C 10.43914000 -7.20926600 -2.66277100
C 9.63075000 -9.47919100 -2.41110000
H 7.49340400 -9.71485000 -2.45142500
C 10.68584700 -8.55301200 -2.49127000
H 11.25482200 -6.49828600 -2.72632700
H 9.84239900 -10.53199000 -2.27611100
H 11.71051300 -8.89741000 -2.41805800
H 9.63527100 -4.77366200 -2.97699000
C 1.96523400 -0.68592600 -0.82355800
N 1.93728700 -2.00696700 -2.79401400
H 4.71205600 -2.48605400 -1.20539800
H 1.45591500 -2.31779100 -3.62606700

s-cis/s-cis

E = -1635.14758860
DG = -1634.765026

0 1
O -0.04734100 0.15528900 0.18426800
C 1.32773300 0.47669400 0.06935100
C 1.44605200 1.94571500 -0.36740500
C 0.75548300 2.81626400 0.67237200
C -0.68736600 2.38649400 0.85037200
C -0.72658000 0.90277300 1.19166100
C 3.40140900 -0.64106800 -0.86410100
N 3.91288800 -1.58644500 -1.73850000
C 3.19998100 -2.34942600 -2.64608600
C 1.24927100 -1.22551900 -1.77003500
N 3.66989900 -3.19220300 -3.48212800
O 4.15591700 -0.01520200 -0.13029200
O 2.77627200 2.38814600 -0.54945800
O 0.75027300 4.17649800 0.28745100
O -1.30927100 3.09420000 1.90335100
C -2.13237800 0.34345900 1.24799100
O -2.11550200 -1.03087900 1.57780200
H 3.38125800 1.77075100 -0.11237700
H 1.66740700 4.42304300 0.12225100
H -1.51024400 -1.45056100 0.95689600
H -1.13434500 4.02942800 1.75001200
H 0.17113400 -1.16206800 -1.82967700
H -2.61867600 0.51488100 0.27900200
H -2.69705300 0.86561200 2.01997400
H 1.82183000 0.35950100 1.04627800
H 0.92503900 2.05387100 -1.32589500
H 1.27733400 2.69633800 1.63400200
H -1.21895900 2.55124800 -0.09795600
H -0.22985500 0.73955400 2.15849000
C 5.04338400 -3.49568900 -3.49382300
C 5.71790300 -4.00554900 -2.39782400
C 5.75021200 -3.35531700 -4.70979800
C 7.07597300 -4.35446900 -2.47601500
H 5.20962600 -4.16437000 -1.45286800
C 7.07950500 -3.68765100 -4.80869700
H 5.21720200 -2.97615900 -5.57341600
C 7.76546000 -4.19232800 -3.68669200
C 7.76063300 -4.89506700 -1.28967500
H 7.60731000 -3.56834900 -5.74781500
N 9.09334800 -4.53017000 -3.77842500
C 9.18024500 -5.23005200 -1.47826100
O 7.18463900 -5.05305000 -0.21961100
C 9.80612400 -5.03702600 -2.72274100
C 9.92775500 -5.75018900 -0.40811700
C 11.16658800 -5.36664200 -2.87874400
C 11.25753200 -6.07104800 -0.56263200
H 9.41913500 -5.88782700 0.53836800
C 11.87310300 -5.87447300 -1.81164500
H 11.64362900 -5.21682200 -3.84042900
H 11.82775900 -6.47086800 0.26586200
H 12.91923300 -6.12497900 -1.94098500
H 9.56390500 -4.40367200 -4.66366700
C 1.95970300 -0.48040700 -0.89836800
N 1.84100100 -2.11372900 -2.61441900
H 4.92146600 -1.69250700 -1.73159200
H 1.28688800 -2.65978700 -3.25904900

Neutral GLAC

s-cis/s-trans

E = -1635.14840797
DG = -1634.766179

0 1
O -0.03608300 0.23079000 0.07384000
C 1.34439900 0.48094900 0.11980200
C 1.61918500 1.91414400 -0.34455100
C 0.85971000 2.85957200 0.57708100
C -0.61792500 2.51655300 0.59362100
C -0.78430900 1.05163600 0.97720600
N 1.99877700 -0.50964000 -0.73636100
C 3.33231800 -0.84511100 -0.44231100
N 3.92314100 -1.81990900 -1.17091700
C 3.27993000 -2.37705600 -2.17983600
C 1.94895200 -2.00298400 -2.55611300
C 1.34608600 -1.06715400 -1.79218200
N 3.88525400 -3.34874700 -2.90484400
O 3.90353500 -0.23828800 0.46496700
O 2.98905900 2.25077600 -0.35942100
O 0.98081700 4.20013100 0.15038300
O -1.31453200 3.29413200 1.54354800
C -2.21648700 0.56992100 0.88855400
O -2.32251000 -0.78303800 1.28007300
H 3.47729600 1.59223300 0.16057100
H 1.92244700 4.40397300 0.12082500
H -1.70835400 -1.27910500 0.72856200
H -1.07165000 4.21304300 1.38358300
H 1.44133800 -2.45575500 -3.39440800
H 0.33397200 -0.72531600 -1.95731800
H -2.57673600 0.71995300 -0.13706500
H -2.83195500 1.16179600 1.56538400
H 1.73522700 0.32733600 1.13167300
H 1.23053400 2.02083900 -1.36468400
H 1.25473100 2.74839700 1.59798600
H -1.02493600 2.67269900 -0.41564000
H -0.40833700 0.89524600 1.99722100
H 3.30971500 -3.79993300 -3.60188000
C 5.16404000 -3.92076000 -2.71789600
C 5.34239700 -5.24599400 -3.06105300
C 6.27093200 -3.17411100 -2.26183000
C 6.59819700 -5.85794900 -2.95758000
H 4.51461800 -5.84644100 -3.42161000
C 7.50695800 -3.76468900 -2.14050700
H 6.14185600 -2.13566600 -1.99870300
C 7.69165900 -5.11645800 -2.48520300
C 6.75441200 -7.27275200 -3.33536000
H 8.35107000 -3.18426600 -1.78657500
N 8.92565800 -5.70493100 -2.36817900
C 8.11005500 -7.82043500 -3.17694700
O 5.81740600 -7.94308300 -3.75156100
C 9.15919100 -7.01622600 -2.69691300
C 8.36915000 -9.16106900 -3.50748800
C 10.44905000 -7.56293600 -2.55528400
C 9.63111900 -9.69355200 -3.36841800
H 7.54180000 -9.75652600 -3.87404800
C 10.67274700 -8.88045700 -2.88786600
H 11.25443700 -6.93931900 -2.18459200

H	9.82429900	-10.72720100	-3.62462500
H	11.66848700	-9.29237600	-2.77553400
H	9.69705600	-5.14726300	-2.02928600

s-cis/s-cis

E = -1635.14886215
DG = -1634.765545

0 1

O	-0.11680300	0.17486500	-0.04704900
C	1.26198700	0.37385900	0.12584400
C	1.57876700	1.86883400	0.03052300
C	0.78198500	2.58732100	1.11158500
C	-0.69833100	2.28738000	0.97012200
C	-0.90470200	0.77794800	0.98515800
N	1.94976900	-0.40047900	-0.90827400
C	3.25764700	-0.83408400	-0.63542400
N	3.87272100	-1.62719000	-1.54479100
C	3.27895500	-1.91241800	-2.68789000
C	1.97902200	-1.41479000	-3.03324900
C	1.34994200	-0.66917500	-2.10064200
N	3.89889200	-2.70566100	-3.59501800
O	3.78948600	-0.47735200	0.41631400
O	2.95175100	2.16434400	0.16278400
O	0.94261400	3.98738400	1.02270100
O	-1.43757600	2.83550900	2.04023400
C	-2.33556000	0.36857800	0.70925500
O	-2.48224100	-1.03514400	0.76420000
H	3.40211600	1.39206200	0.54056100
H	1.88656600	4.16924200	1.09287300
H	-1.84465700	-1.40439700	0.14406200
H	-1.17448400	3.75963700	2.11608100
H	1.51100500	-1.63902200	-3.98011600
H	0.35556300	-0.26622400	-2.23177900
H	-2.63370300	0.76498900	-0.26961800
H	-2.98123400	0.80102500	1.47302600
H	1.59671500	-0.02525500	1.08964300
H	1.24710200	2.22470200	-0.95251000
H	1.11842400	2.22782600	2.09558300
H	-1.04596700	2.68829100	0.00723100
H	-0.59016100	0.37476500	1.95718400
H	3.37960300	-2.86423500	-4.44697300
C	5.15482000	-3.34114300	-3.55242000
C	6.03244800	-3.31375100	-2.48412900
C	5.51738300	-4.06431100	-4.71228600
C	7.25981200	-3.99274400	-2.55856300
H	5.79903200	-2.77494700	-1.58080600
C	6.71205200	-4.73260000	-4.79689000
H	4.83627800	-4.09199700	-5.55607100
C	7.60969800	-4.70712700	-3.71244900
C	8.17504400	-3.94468100	-1.40445300
H	6.96821900	-5.27961700	-5.69641100
N	8.80866900	-5.37123200	-3.78484500
C	9.43726100	-4.68283700	-1.56747600
O	7.90286700	-3.33138700	-0.37951800
C	9.71754900	-5.37651300	-2.75801400
C	10.38004800	-4.69995200	-0.52599200
C	10.93339700	-6.07466000	-2.89022500
C	11.56802400	-5.38340400	-0.65682100
H	10.13690800	-4.15701900	0.37928800
C	11.83807900	-6.07310900	-1.85203800
H	11.14466400	-6.60653200	-3.81079300
H	12.28992900	-5.39240000	0.14953300
H	12.77097100	-6.61284800	-1.96266000
H	9.03003500	-5.88043300	-4.62906100

s-trans/s-trans

E = -1635.14745719
DG = -1634.764979

0 1

O	0.10562300	0.18892200	0.18332400
C	1.50179200	0.19857200	0.03926000
C	1.98404800	1.63890300	-0.15586800
C	1.55418800	2.44581700	1.06237900
C	0.05403400	2.34810800	1.26463800
C	-0.34220400	0.87984300	1.35460600
N	1.83212600	-0.65066500	-1.10680100
C	3.10775400	-1.25062000	-1.12694600
N	3.39674300	-2.09599300	-2.13649500
C	2.52508800	-2.29543900	-3.11573800
C	1.24536300	-1.65857500	-3.15654200
C	0.94516200	-0.84947000	-2.11427100
N	2.91032600	-3.16533700	-4.07667900
O	3.89839800	-0.96962400	-0.22338800
O	3.37806700	1.74927900	-0.34016300
O	1.87511600	3.81266300	0.91200900
O	-0.34459700	2.97756400	2.46339900
C	-1.83989600	0.66916200	1.41495700
O	-2.15610900	-0.70307800	1.52379400
H	3.79450500	0.91405800	-0.07137000
H	2.82588500	3.86520500	0.76253500
H	-1.72886000	-1.14473700	0.78229600
H	0.04975500	3.85699700	2.46086100
H	0.52836500	-1.83789600	-3.94123900
H	-0.00520800	-0.34388100	-2.01409600
H	-2.29560900	1.11615600	0.52234600
H	-2.23304700	1.17273600	2.29765000
H	1.98762200	-0.25117500	0.91207700
H	1.48768200	2.04423100	-1.04613400
H	2.05404800	2.03354900	1.95175600
H	-0.44601800	2.80284300	0.39747700
H	0.12678000	0.42687500	2.23845600
H	3.78500400	-3.63958700	-3.89239700
C	2.21390400	-3.48123800	-5.27066900
C	1.98324900	-4.79749900	-5.60652800
C	1.80549600	-2.46015100	-6.15143200
C	1.34363900	-5.12873900	-6.80901700
H	2.29401200	-5.60212100	-4.95072100
C	1.15169000	-2.76160000	-7.32206500
H	2.01807900	-1.42676100	-5.90363900
C	0.91239600	-4.10570200	-7.66899500
C	1.11836200	-6.54450500	-7.14985800
H	0.83656700	-1.97151100	-7.99313400
N	0.27256200	-4.41503000	-8.84049900
C	0.42585700	-6.78223000	-8.42539500
O	1.48424400	-7.45572700	-6.41874600
C	0.02272300	-5.70651900	-9.23561200
C	0.16178800	-8.09577800	-8.84694000
C	-0.63725800	-5.95835900	-10.45283100
C	-0.48424700	-8.34024800	-10.03803300
H	0.48440000	-8.90412800	-8.20212700
C	-0.88297400	-7.25713700	-10.84033200
H	-0.94566900	-5.12544200	-11.07396300
H	-0.68504400	-9.35460400	-10.35758500
H	-1.39146700	-7.44156400	-11.77903800
H	-0.02513800	-3.65975200	-9.44251600

s-trans/s-cis

E = -1635.14748221
DG = -1634.764598

0 1

O	0.08745200	0.09791300	0.04572400
C	1.48912000	0.14865300	-0.00633900
C	1.94386600	1.60930000	-0.07415200
C	1.41649100	2.31977100	1.16580300
C	-0.09052400	2.17673400	1.26314600
C	-0.45333100	0.69754200	1.22791500
N	1.91305300	-0.61649200	-1.18066700
C	3.20231300	-1.18537100	-1.15718800
N	3.57230000	-1.96445900	-2.19326300
C	2.76638100	-2.12761900	-3.23386600
C	1.47687700	-1.51582000	-3.31736300
C	1.09371900	-0.77695200	-2.25040200

N	3.23362900	-2.94031200	-4.20972900
O	3.93028600	-0.94075400	-0.19223700
O	3.34323100	1.76406200	-0.15849700
O	1.71195000	3.70002900	1.13134000
O	-0.57977800	2.71298300	2.47369700
C	-1.94560400	0.44975200	1.17341300
O	-2.23124700	-0.93352400	1.17218400
H	3.76409200	0.92054300	0.07572400
H	2.66846000	3.78219100	1.04480200
H	-1.74290000	-1.31579200	0.43542000
H	-0.21140600	3.59997500	2.55439200
H	0.81347300	-1.66215900	-4.15375500
H	0.12593800	-0.30024700	-2.18071800
H	-2.35298700	0.94405700	0.28236700
H	-2.41001500	0.88491300	2.05782100
H	1.93062800	-0.34659700	0.86542700
H	1.49392900	2.06345700	-0.96550400
H	1.86930300	1.85594100	2.05511800
H	-0.54636300	2.67995200	0.39848800
H	-0.03084900	0.19586200	2.10884200
H	4.10765900	-3.39954400	-3.98634700
C	2.63550600	-3.22152100	-5.46205700
C	2.18782600	-2.21910600	-6.29629200
C	2.53709200	-4.56316300	-5.87932900
C	1.60165300	-2.52597000	-7.53195900
H	2.27926700	-1.17471700	-6.02205600
C	1.99413700	-4.88381000	-7.10032900
H	2.89501400	-5.34764300	-5.22300500
C	1.50747500	-3.86543800	-7.94285400
C	1.11262300	-1.43673100	-8.39573700
H	1.92499800	-5.91822100	-7.41522400
N	0.94808500	-4.17579700	-9.15489500
C	0.52800800	-1.86270800	-9.67606500
O	1.18840100	-0.26053400	-8.06446100
C	0.46169800	-3.22474300	-10.01763800
C	0.02535800	-0.90499800	-10.57237500
C	-0.10552400	-3.60961500	-11.24684600
C	-0.52823300	-1.28335900	-11.77491600
H	0.08995900	0.13669200	-10.28230300
C	-0.59004800	-2.64821100	-12.10591400
H	-0.15597600	-4.66124500	-11.50414500
H	-0.91378700	-0.54094200	-12.46158100
H	-1.02520800	-2.95361800	-13.04990700
H	0.89500500	-5.14724200	-9.42846700

s-cis/s-trans with conformationally locked dihedrals at ψ_1, ψ_2

E = -1635.14814631

0 1

O	-0.97128490	-3.75303190	-6.27473258
C	-0.41773490	-4.88153551	-5.64993536
C	-1.48332352	-5.56162211	-4.78610043
C	-2.64375752	-5.95107735	-5.69251699
C	-3.17121361	-4.73785576	-6.43501772
C	-2.02379421	-4.07793834	-7.18926011
N	0.72527738	-4.42695846	-4.85694088
C	1.75622829	-5.34920244	-4.61119896
N	2.85950011	-4.91733632	-3.95636072
C	2.91976566	-3.68214159	-3.49561766
C	1.84843890	-2.74229384	-3.65482452
C	0.77394652	-3.16250383	-4.35513088
N	4.02490400	-3.25159000	-2.84075600
O	1.61438446	-6.50887318	-5.00082702
O	-1.00916064	-6.69537769	-4.09326171
O	-3.71463537	-6.49480944	-4.95009085
O	-4.15553908	-5.10146022	-7.37901393
C	-2.41618014	-2.77443222	-7.85122747
O	-1.32827751	-2.21602086	-8.55799055
H	-0.16754295	-6.97344183	-4.48883450
H	-3.36670132	-7.25048592	-4.46329357
H	-0.60352556	-2.13370063	-7.92915709
H	-4.79709424	-5.65148533	-6.91551941
H	1.90077149	-1.74023480	-3.25632812
H	-0.08007963	-2.53510622	-4.56849059
H	-2.79012565	-2.08668616	-7.08219032
H	-3.21513004	-2.96668241	-8.56672079
H	-0.02391423	-5.58488231	-6.39170533
H	-1.83708580	-4.83322565	-4.04623345
H	-2.28301236	-6.68228482	-6.43133737
H	-3.57560213	-4.02659470	-5.70062357
H	-1.63716002	-4.77195360	-7.94778073
H	3.95595389	-2.31840452	-2.45935531
C	5.28128698	-3.86103148	-2.64617821
C	6.12493272	-3.27755390	-1.71688897
C	5.70904297	-5.02924003	-3.31414490
C	7.38276519	-3.81921177	-1.43384904
H	5.83248633	-2.38142259	-1.17999193
C	6.94745615	-5.56894784	-3.04704638
H	5.06251353	-5.49311788	-4.04111204
C	7.80583320	-4.97627066	-2.10592517
C	8.25073426	-3.16775072	-0.43731249
H	7.26463400	-6.46533890	-3.56738179
N	9.04002735	-5.51680201	-1.84185296
C	9.55264424	-3.81478821	-0.21846756
O	7.90719360	-2.15741983	0.16407561
C	9.90917158	-4.97459374	-0.93014991
C	10.45747597	-3.27410829	0.71074140
C	11.16241699	-5.57493001	-0.70137903
C	11.68203256	-3.86263364	0.93210888
H	10.15496153	-2.38052221	1.24316175
C	12.02815962	-5.02202094	0.21561720
H	11.43321987	-6.46859084	-1.25154151
H	12.37449489	-3.44104686	1.64913118
H	12.99002245	-5.49129722	0.38426347
H	9.31893930	-6.35029778	-2.34041514

Table S3: Kinetic studies for the calculation of the % inhibition of GPMM activity in the presence of compounds **4**, **5**, **6**

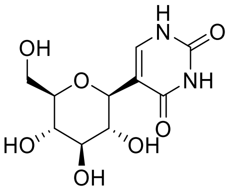<br>**4**<br>[**I**]: 20, 50, 100, 150, 200 μM<br>IC_{50} = 88.5 ± 4.3 (μM)	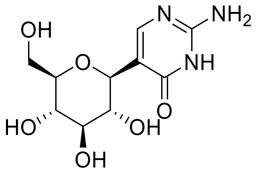<br>**5**<br>[**I**]: 50, 200, 300, 500 μM<br>IC_{50} = 395.7 ± 10.8 (μM)
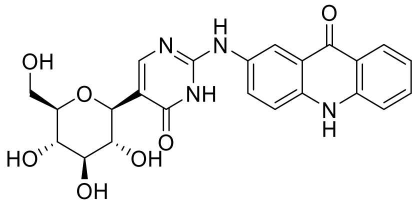<br>**6**<br>[**I**]: 0.5, 1, 5, 10, 20 μM<br>IC_{50} = 5.4 ± 0.5 (μM)	
